# *Hericium erinaceus* mycelium and its small bioactive compounds promote oligodendrocyte maturation with an increase in myelin basic protein

**DOI:** 10.1038/s41598-021-85972-2

**Published:** 2021-03-22

**Authors:** Hui-Ting Huang, Chia-Hsin Ho, Hsin-Yu Sung, Li-Ya Lee, Wan-Ping Chen, Yu-Wen Chen, Chin-Chu Chen, Chung-Shi Yang, Shun-Fen Tzeng

**Affiliations:** 1 Department of Life Sciences, College of Bioscience and Biotechnology, National Cheng Kung University, #1 University Road, Tainan, Taiwan; 2 Grape King Biotech Research Institute, Zhongli, 320 Taiwan; 3 Institute of Biomedical Engineering and Nanomedicine, National Health Research Institutes, Zhunan Town, Miaoli County, Taiwan

**Keywords:** Nutrition, Glial biology

## Abstract

Oligodendrocytes (OLs), myelin-producing glia in the central nervous system (CNS), produce a myelin extension that enwraps axons to facilitate action potential propagation. An effective approach to induce oligodendrogenesis and myelination is important to foster CNS development and promote myelin repair in neurological diseases. *Hericium* (*H.*) *erinaceus*, an edible and culinary-medicinal mushroom, has been characterized as having neuroprotective activities. However, its effect on OL differentiation has not yet been uncovered. In this study using oligodendrocyte precursor cell (OPC) cultures and an ex vivo cerebellar slice system, we found that the extract from *H. erinaceus* mycelium (HEM) not only promoted the differentiation of OPCs to OLs in the differentiation medium, but also increased the level of myelin basic protein (MBP) on neuronal fibers. Moreover, daily oral administration of HEM into neonatal rat pups for 7 days enhanced MBP expression and OLs in the corpus callosum of the postnatal rat brain. The effect of HEM-derived bioactive compounds, the diterpenoid xylosides erinacine A (HeA) and HeC and a sesterterpene with 5 isoprene units called HeS, were further evaluated. The results showed that HeA and HeS more potently stimulated MBP expression in OLs and increased the number of OLs. Moreover, overlap between MBP immunoreactivity and neuronal fibers in cultured cerebellar tissue slices was significantly increased in the presence of HeA and HeS. In summary, our findings indicate that HEM extract and its ingredients HeA and HeS display promising functional effects and promote OL maturation, providing insights into their potential for myelination in neurodevelopmental disorders.

## Introduction

Oligodendrocytes (OLs) in the central nervous system (CNS) are responsible for myelination, the process in which a compact myelin sheath that surrounds the axon segments, producing effective action potential propagation called saltatory conduction, is formed. The neuronal supporting functions of OLs also include the provision of neurotrophic factors and energy to neurons^1,2^. Myelinating OLs are generated from oligodendrocyte precursor cells (OPCs) derived from neural stem cells (NSCs) through the progression of OL lineages in the developing CNS, and OPCs that reside in the subventricular zone (SVZ) are the major resource of oligodendrogenesis in the adult CNS^3,4^. In general, myelination robustly occurs after birth, and continues in adult^5^. Early myelination during CNS development can be observed in the hindbrain and midbrain at 3 month and postnatal day 7 (P7) in human and rat, respectively; lastly, it occurs in the corpus callosum and cerebellum at 6 month in human and P10 in rat^5–7^. In the rodent model, few OLs can be observed at P3 through immunostaining of myelin basic protein (MBP), a critical myelin protein to maintain myelin compaction^8^. In addition, MBP-immunostained fibers are able to be detected in the cingulum, somatosensory cortex, and the corpus callosum of mice at P7^8^. Given that myelination is important for memory in new skill learning, movement and cognition^9,10^, the dysregulation of OL lineage progression is one of causes of neurodevelopmental disorders, such as autistic-like syndrome^11,12^, as well as demyelinating diseases, such as multiple sclerosis^13^. Thus, the characterization of functional therapeutic agents that stimulate the differentiation of OPCs into mature OLs is of interest for the treatment of neurodevelopmental disorders.

*Hericium* (*H.*) *erinaceus,* commonly called lion’s mane, is an edible and medicinal mushroom widely used in Asia. The mycelium and fruiting bodies of *H. erinaceus* contain many bioactive compounds with good pharmaceutical efficacy^14,15^. Erinacines (cyathane diterpenoids), constituents of *H. erinaceus* mycelium (HEM), can pass through the blood–brain barrier^15,16^. Among the 15 erinacines (HeA-K and P-S), HeA-I has been found to induce the production of nerve growth factor (NGF) to stimulate neuronal differentiation^15,17–19^. HeA-enriched HEM extract was reported to reduce Aβ plaques and gliosis in the cortex and hippocampus of 5-month-old female APPswe/PS1dE9 transgenic mice and increase NGF and hippocampal neurogenesis^20^. The findings also indicated that HeS, a sesterterpene isolated from HEM ethanol extract, attenuated Aβ plaque accumulation in 5-month-old female APP/PS1 transgenic mice and increased the level of insulin-degrading enzyme in the cerebral cortex^21^. Treatment with HEM and HeA was found to prevent dopaminergic neurons from neurotoxin MPTP (1-methyl-4-phenyl-1,2,3,6-tetrahydropyridin)-induced cell death^22^, and exerted an anti-inflammatory protective effect against ischemic injury in neurons^23^. HeC also had an inhibitory effect on iNOS-associated inflammation in the mouse BV2 microglial cell line^24^. These findings demonstrate that crude HEM and its active substances (i.e., HeA, HeC, and HeS) can regulate the function of CNS neurons, astrocytes and microglia via either NGF activity or anti-inflammatory effects.

To make HEM a potential function food, in addition to neuroprotective effects of erinacines, it is also of great interest to evaluate their action on OL differentiation and myelination. Thus, in this study, we examined the bioactivity of crude HEM and its three bioactive compounds (HeA, HeC, and HeS) in stimulating OPC differentiation into mature OLs with the expression of myelin proteins, MBP and myelin proteolipid protein (PLP). The action of the crude HEM on the induction of myelin production in the corpus callosum of the developing neonatal rat brains was validated. The in vitro and ex vivo results also provide significant evidence of the promising efficacy of HEM-derived components (HeA and HeS) in promoting OPC differentiation into mature OLs.

## Results

### Treatment with HEM extract increased OL differentiation

**Figure 1:**
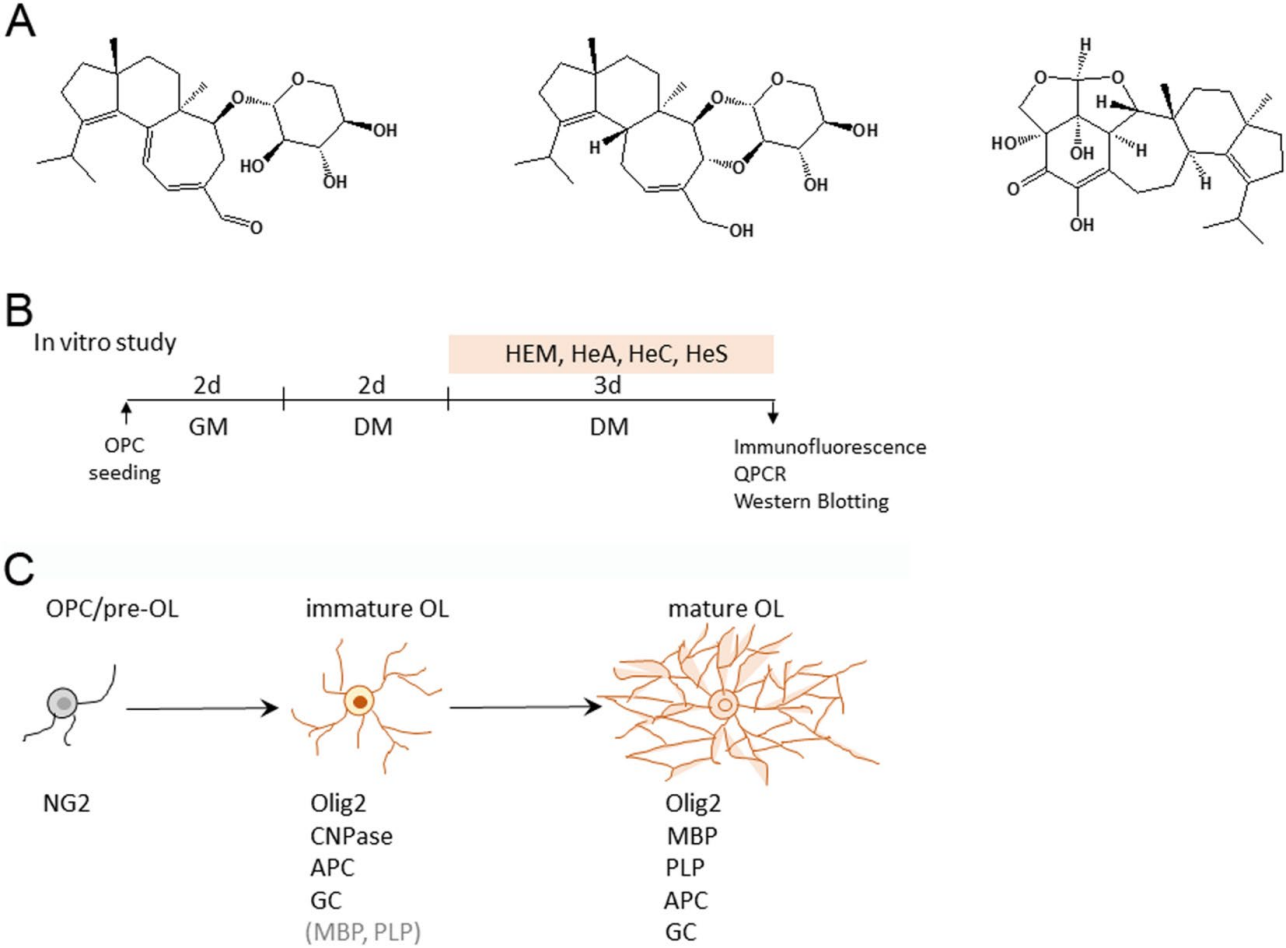
Schematic diagram of the experimental designs. (**A**) The structures of HeA, HeC, and HeS from the *H. erinaceus* mycelium (HEM) are depicted. **(B**) In an in vitro experiment, OPCs were seeded in GM for 2 days and then grown in DM for another 2 days. Then, crude HEM, HeA, HeC, or HeS at the indicated concentrations was added to the cultures maintained in DM for 3 consequential days. The cultures were subjected to immunofluorescence to detect GC and MBP, Western blot analysis to measure MBP and PLP, and qPCR to measure OL differentiation-associated genes. (**C**) The cell markers used to identify OL lineages are illustrated. In this study, immunostaining for NG2, APC, GC, and myelin proteins (MBP and PLP) was conducted to identify OPCs and OLs. Note that the detectable level of MBP and PLP immunoreactivity can be observed in immature OLs in culture.

**Figure 2:**
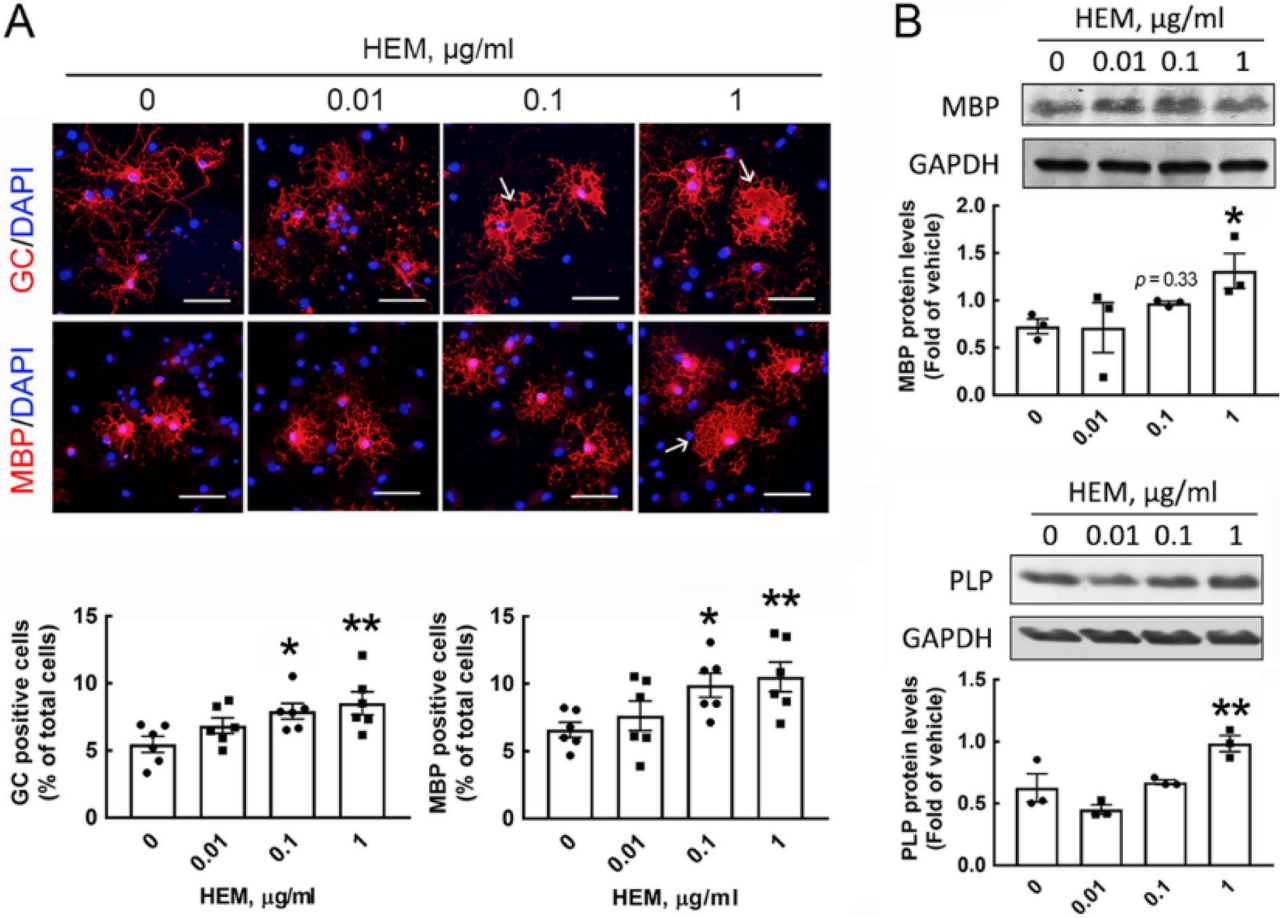
Crude HEM enhanced OL maturation. Crude HEM at the indicated concentrations was added to the cultures for 3 days after OPCs had been maintained in DM for 2 days (Fig. 1B). (**A**) The cultures were subjected to GC and MBP staining (red), followed by DAPI nuclear counterstaining (blue). GC^+^- and MBP^+^-OLs in the cultures were quantified as described in the “Materials and methods” section. Each spot represents as the data quantified from one photo captured from the cultures. Arrows shown in the representative images indicate OLs with the shape of an extended membrane. (**B**) Total proteins were isolated from cultures treated with crude HEM for 3 days and subjected to Western blot analysis to examine MBP and PLP protein levels. The intensity of the immunoreactive bands shown in the immunoblots was quantified by ImageJ software version 1.52a (https://imagej.nih.gov/ij/) and normalized to the level of GAPDH, which was used as a loading control. The immunoblot images used for quantification are provided in Fig. S5. Data are presented as the means ± SEMs of the three independent experiments. The raw immunoblot images are shown in Fig. S5. *p < 0.05, **p < 0.01 compared with the control culture. Scale bar in (**A**) 50 μm.

**Figure 3:**
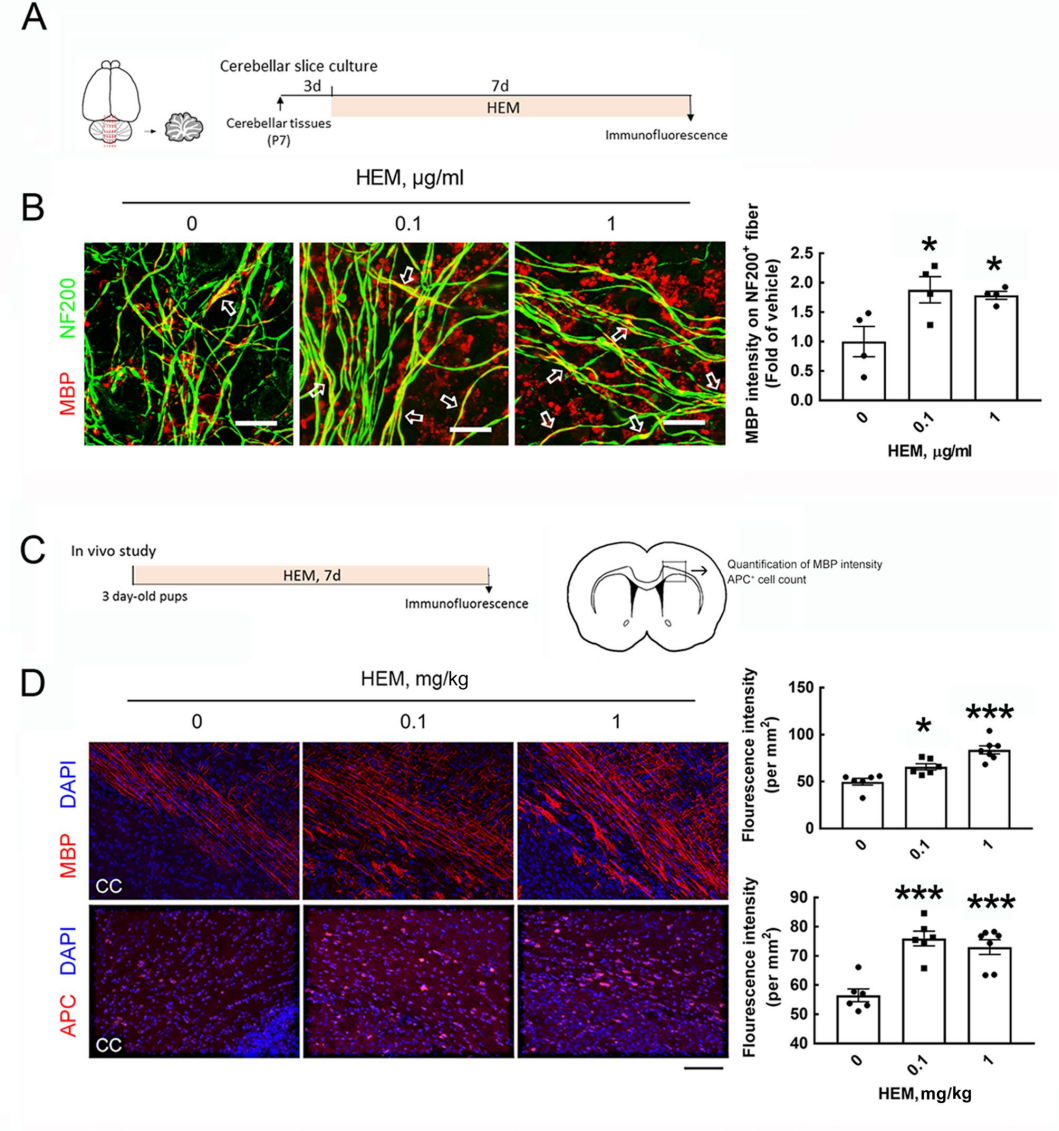
Ex vivo and in vivo experiments reveal the effect of crude HEM in stimulating MBP production and OL maturation. (**A**) Cerebellar tissue slices taken from the vermis (as indicated by red lines) were prepared from P7 rats and then cultured for 3 days. Crude HEM at concentrations of 0.1 and 1 μg/ml was added to the cultures for 7 days. The cultures were subjected to immunofluorescence for NF200 and MBP staining. (**B**) Confocal images show overlap between the immunoreactive intensity of MBP with NF200^+^-fibers (arrows), which was quantified using ImageJ software. The data were obtained from four random fields per culture (right panel). The results are presented as the means ± SEMs of the four tissue slices for each treatment. Each dot denotes data obtained from one tissue slice. (**C**) For the in vivo study, crude HEM was administered to rat pups at the age of P3 orally daily for 7 consecutive days. After brains were removed and cryostat sectioned, followed by immunofluorescence for MBP or APC. MBP intensity and APC+-cells were quantified in the body portion of the corpus callosum using ImageJ software. (**D**). After immunofluorescence by anti-MBP (red) or anti-APC (red), the brain tissue sections were subjected to DAPI nuclear counterstaining (blue). The representative images in the body portion of the corpus callosum were captured by confocal microscopy, and the immunoreactive intensity of MBP and APC was then quantified as described in “Materials and methods” section. The results are presented as the means ± SEMs of 6 animals in each group. *p < 0.05, ***p < 0.001 versus the vehicle-treated control culture. Scale bar in (**B**) 20 μm; in (**D**) 50 μm.

The cell viability of OPCs cultured in GM was examined after treatment with different concentrations of HEM extract for 24 and 48 h. The results showed that crude HEM caused a nonsignificant reduction in the cell survival of OPCs (Fig. S1). Further in vitro experiments were designed to determine the effect of HEM on OL differentiation, as shown in Fig. 1B. GC immunofluorescence was conducted to identify immature OLs/mature OLs in cultures, whereas myelin proteins (i.e. MBP and PLP) expression was examined to determine OL maturation (Fig. 1C). When OPCs were cultured in DM for 48 h, we found that the gene expression of the OL-specific transcriptional factor Olig2 or myelin proteins after exposure to 0.1 and 1.0 μg/ml crude HEM for 3 days tended to increase (Fig. S2A). Exposure to 0.1 and 1.0 μg/ml crude HEM for 3 days significantly increased the numbers of GC^+^- and MBP^+^-OLs dose-dependently (Fig. 2A). Moreover, these immunostained OLs in the cell cultures treated with 0.1 and 1.0 μg/ml crude HEM displayed a mature morphology with a complex and extended membranous shape (Fig. 2A, arrows). This was in accord to the results showing an increased level of MBP protein expression after exposure to crude HEM at 0.1 and 1.0 μg/ml in DM (Fig. 2B). The addition of crude HEM at the dose of 1 μg/ml also increased PLP protein levels in the cultures. Since MBP immunostaining is an ideal approach to evaluate myelination in vivo^8^, we further used an ex vivo rat cerebellar tissue slice culture system to examine neuronal fibers covered by MBP^+^-OL cellular processes (Fig. 3A). The results showed that overlap between MBP immunoreactivity and neuronal fibers was significantly higher in crude HEM-treated cultures than in the control culture (Fig. 3B, arrows). An in vivo experiment was conducted via the daily oral administration of HEM extract at 0.1 and 1.0 mg/kg to P3 rat pups for 7 days (Fig. 3C). The results indicated that MBP immunoreactivity was much more intense in the body of the corpus callosum of rat pups than in that of the vehicle-treated control rat pups (Fig. 3D). The data by quantification of adenomatous polyposis coli (APC)^+^-OLs located in the body portion of the corpus callosum also revealed that OLs were increased in number after the oral intake of crude HEM for 7 days (Fig. 3D). Accordingly, these findings show the beneficial effect of crude HEM in promoting OL maturation and myelination in the white matter of developing brains.

### The HEM-derived bioactive compounds HeA and HeS efficiently improve OL maturation

**Figure 4:**
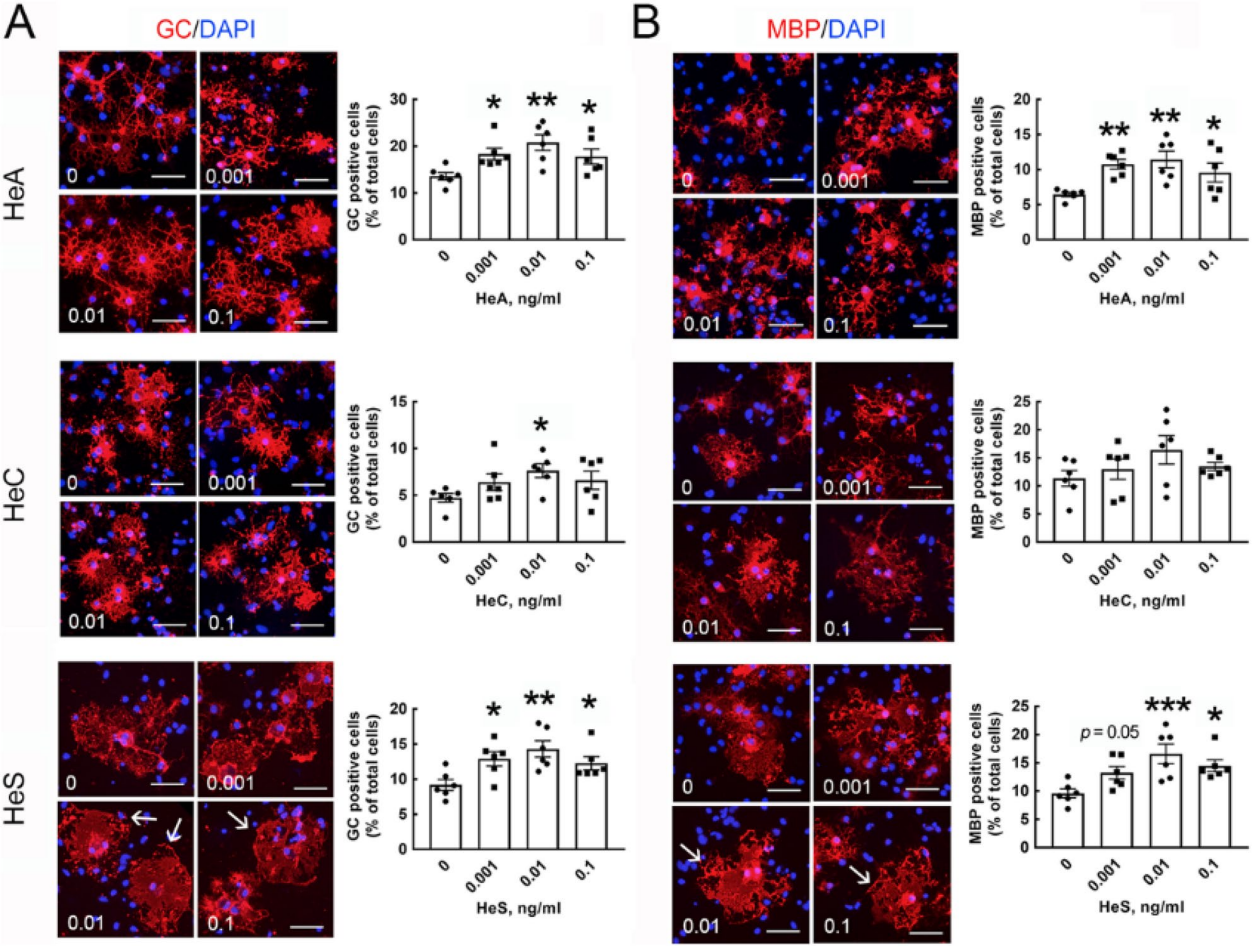
Three HEM-derived compounds promote OL maturation. After OPCs were maintained in DM for 2 days, as described in Fig. 1B, three known HEM-derived compounds (HeA, HeC, HeS) were added to the cultures at the indicated concentrations and incubated for another 3 days. The cultures were processed for GC (**A**) and MBP (**B**) immunofluorescence (red) and DAPI nuclear counterstaining (blue). The OLs with a membranous extension are indicated by arrows in the representative images. GC^+^- and MBP^+^-OLs in the cultures were quantified as described in “Materials and methods” section. Each spot represents as the data quantified from one photo captured from the cultures. The experiments were repeated by the three independent cell preparations. *p < 0.05, **p < 0.01, ***p < 0.001 versus the vehicle-treated control culture. Scale bar, 50 μm.

**Figure 5:**
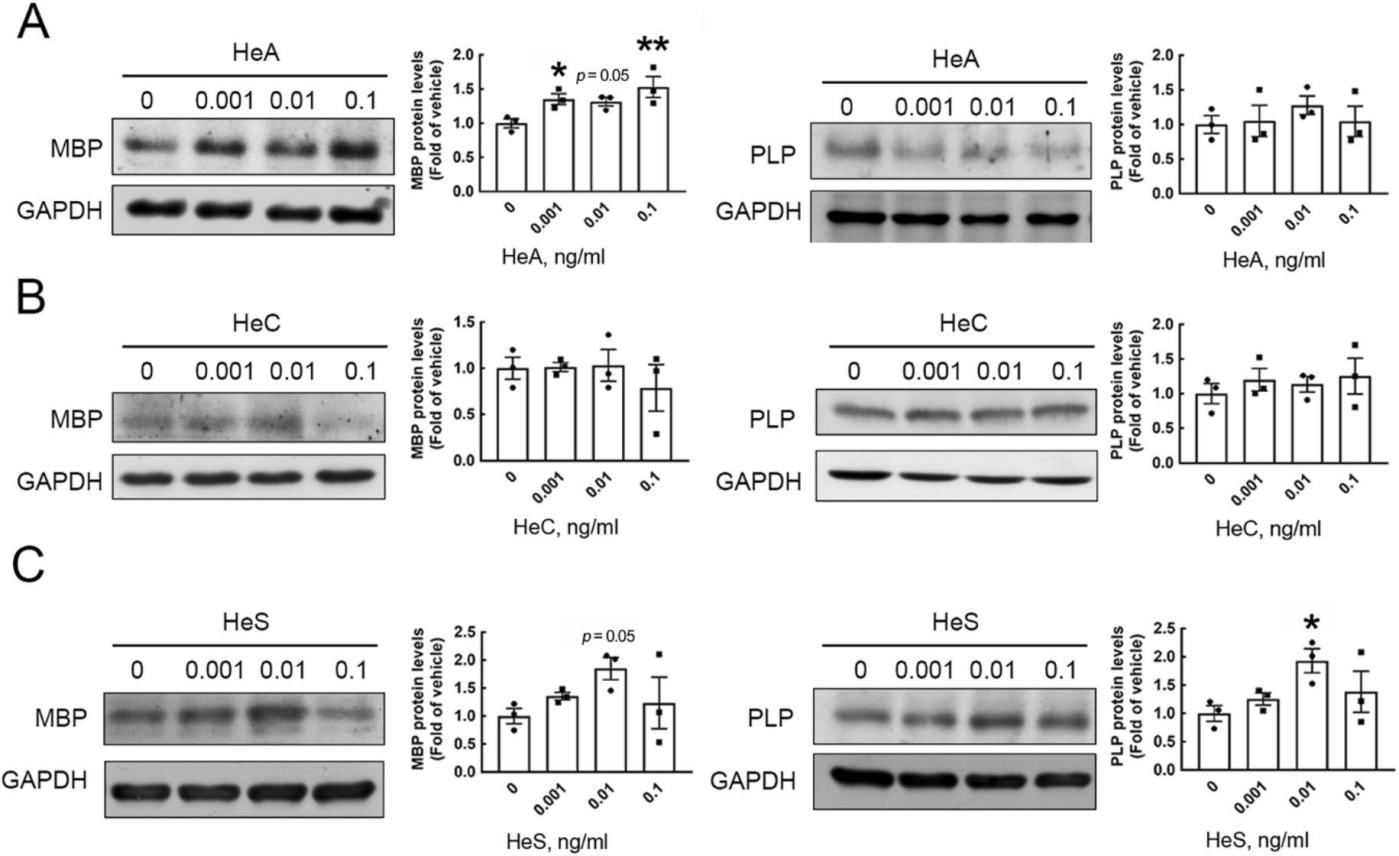
HeA and HeS increased MBP protein production in OL cultures. OPCs were first maintained in DM for 2 days and then exposed to HeA, HeC, or HeS at the indicated concentrations for another 3 days (Fig. 1B). Total proteins were extracted from the cultures and subjected to Western blot analysis to examine MBP (**A**) and PLP (**B**) protein levels. The GAPDH level was used as a loading control. The intensity of the immunoreactive bands shown in the immunoblots was quantified by ImageJ software version 1.52a (https://imagej.nih.gov/ij/), and normalized to the level of GAPDH, which was used as a loading control. The results are presented as the means ± SEMs of three repeated experiments. The raw immunoblot images are shown in Figs. S6, S7, and S8. *p < 0.05, **p < 0.01 compared with the control culture.

Among the known components of HEM extract, HeA, HeC, and HeS have been purified and characterized as the neuroprotective and anti-inflammatory compounds^15,21,24^. Thus, we further evaluated the effect of HeA, HeC, and HeS in stimulating OL maturation. Treatment of OPCs in GM with the three components at concentrations from 0.001 to 10 ng/ml did not affect OPC viability (Fig. S1). Through examination of OL differentiation-associated genes by QPCR, the three compounds at 0.1, 1.0, and 10 ng/ml to OPCs in GM insignificantly changed the expression of Olig1, Olig2, MBP, and PLP (Fig. S2B–D). However, when OPCs were maintained in DM, treatment with HeA or HeS from 0.001 to 0.1 ng/ml for 3 days increased the number of GC- and MBP^+^-OLs in the cultures (Fig. 4A, B). Although treatment with HeC at only 0.01 ng/ml did increase in the number of GC^+^-OLs (Fig. 4A), exposure to HeC did not change the number of MBP^+^- OLs in the cultures (Fig. 4B). We also noticed that HeS exposure induced the generation of a membranous extension by OLs (Fig. 4, arrows). Western blot analysis indicated that HeA and HeS increased the production of MBP (Fig. 5A, C), whereas MBP protein levels were not altered by HeC at the different concentrations (Fig. 5B). Notably, an increased level of the PLP protein was detected in the culture treated with 0.01 ng/ml HeS (Fig. 5C), whereas HeA and HeC had no effect on production of the PLP protein. These results reveal that HeA and HeS effectively promote OL maturation. Altogether, these results show that HeA and HeS had a stimulatory effect on OL differentiation via the upregulation of MBP expression.

### An ex vivo experiment shows that HeA and HeS upregulate MBP expression

**Figure 6:**
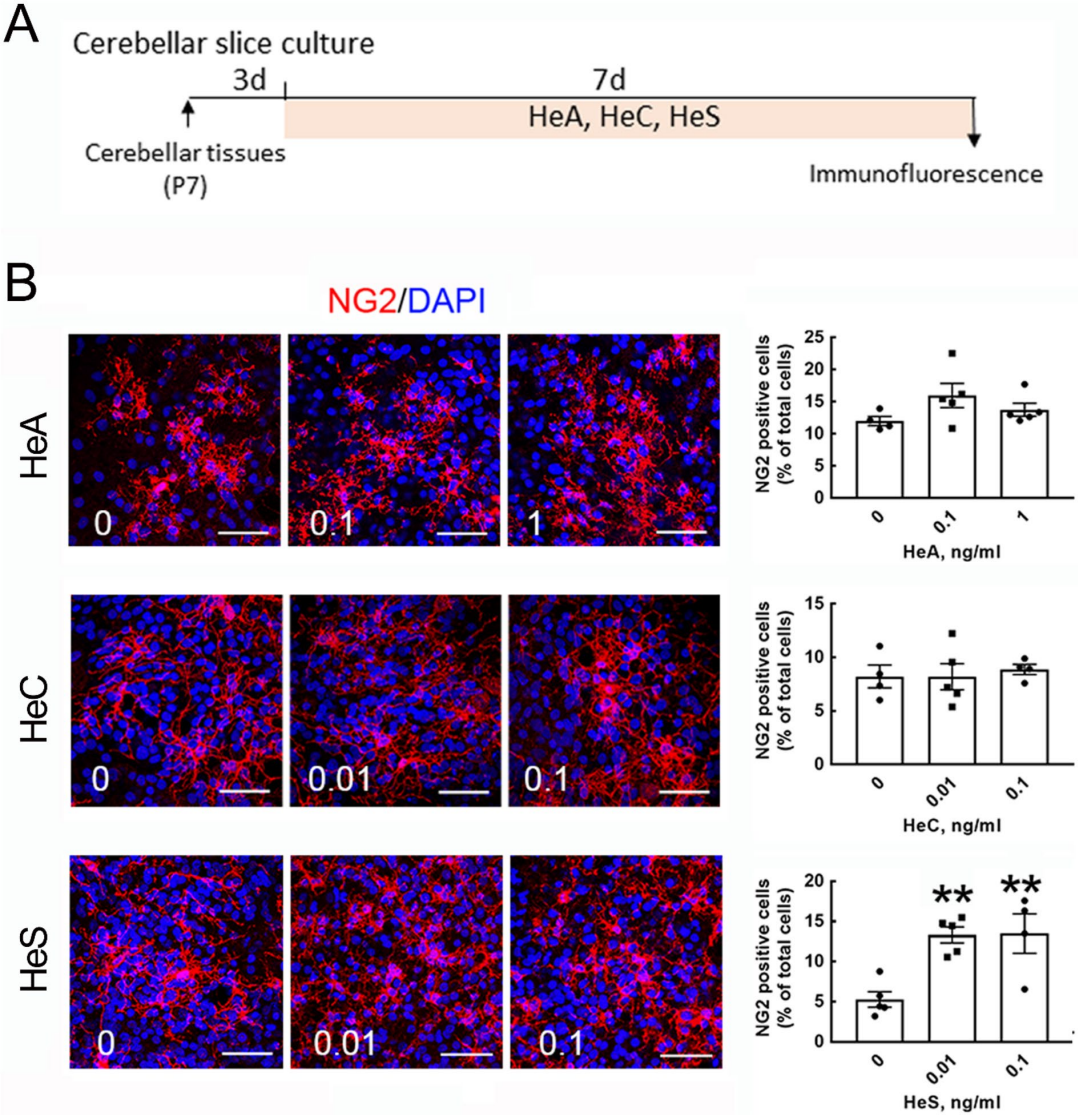
Exposure to HeS increased the number of NG2^+^-OPCs in cerebellar tissue slices. (**A**) The schematic diagram depicts that HeA, HeC, and HeS at the indicated concentrations were added to cerebellar slice cultures and incubated for 7 days. (**B**) The cultures were subjected to NG2 immunofluorescence (red) and DAPI nuclear counterstaining (blue). The representative images captured from confocal microscopy (left panel) and NG2^+^-OPCs in 4 random fields per culture were quantified as described in “Materials and methods” section (right panel). The results are presented as the means ± SEMs of the total tissue slices (n = 4–5) for each treatment. Each dot denotes data obtained from one tissue slice. **p < 0.01 compared with the corresponding control culture. Scale bar, 50 μm.

**Figure 7:**
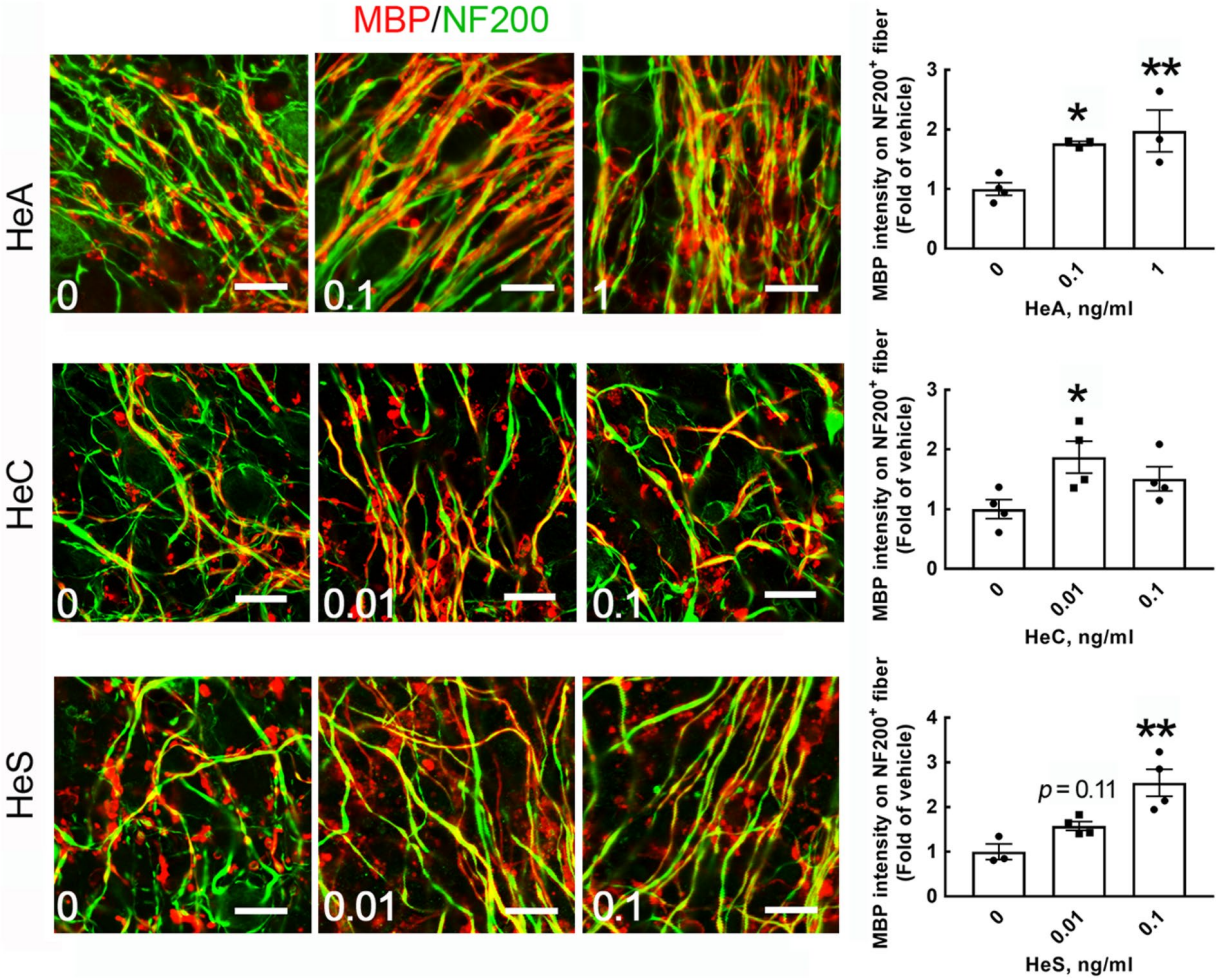
HeA and HeS enhanced the ensheathment of neuronal fibers with MBP^+^-cell processes. As described in Fig. 6A, cerebellar tissue slices were treated with HeA, HeC, or HeS at the indicated concentrations for 7 days. NF200 (green) and MBP (red) double immunofluorescence was conducted. Representative images were captured by confocal microscopy, and the overlap between the MBP signal and NF200^+^-neuronal fibers in 4 random fields per culture was quantified as described in “Materials and methods” section (right panel). The results are presented as the means ± SEMs of three repeated experiments. *p < 0.05, **p < 0.01 compared with the corresponding control culture. Scale bar, 20 μm.

Further experiments in an ex vivo cerebellar slice culture system were performed to examine whether exposure to HeA, HeC, or HeS enhanced MBP expression on the neuronal fibers (Fig. 6A). Because HeA is enriched in crude HEM, as indicated in the “Materials and methods” section, the concentrations of HeA added to the cultures were higher than those of HeC and HeS. As shown in Fig. 6B, the number of NG2^+^-OPCs was increased in the cerebellar slice cultures treated with HeS at 0.01 and 0.1 ng/ml, but not HeA or HeC at any concentration tested in this experiment. Yet, no changes in the amount of APC^+^-OLs in the cerebellar slice cultures were observed after the addition of HeA, HeC, or HeS when compared to that in the control culture (Fig. S3A). The amount of endogenous Olig2^+^-OLs was also not increased in the cultures after treatment with HeA, HeC, or HeS (Fig. S3B). However, as shown in Fig. 7, the intensity of MBP immunoreactivity associated with NF200^+^-neuronal fibers was significantly higher in the slice cultures treated with HeA (0.1 and 1 ng/ml) and HeS (0.1 ng/ml). Note that MBP immunoreactivity covering neuronal fibers only showed a tendency to be increased in HeC-treated cultures.

## Discussion

This is the first study to demonstrate that crude HEM prepared from *H. erinaceus* mycelium containing bioactive ingredients can increase the number of mature OLs in culture and enhance myelination in the corpus callosum of postnatal rat brains. Moreover, our evidence indicates that the HEM derived compounds, HeA and HeS, act as active small molecules to promote OL maturation in culture and myelination in ex vivo cerebellar slice culture.

*H. erinaceus* mycelium has been shown to consist of the functional ingredients that can prevent ischemic stroke, Parkinson's disease (PD), Alzheimer's disease (AD), and depression^15,19,20,25^. Here, we show that daily oral administration of HEM extract to neonatal rat pups at P3 for 7 days promoted MBP protein expression and increased the number of immature/mature OLs in the corpus callosum at P10, demonstrating that HEM contains functional constituents to promote OL maturation and myelination during the white matter formation in the postnatal rat brain. The in vitro evidence showed the positive effect of crude HEM and its compounds in promoting OL maturation, which substantiates the potential use of HEM and its small active compounds for the treatment of neurodegeneration-associated demyelination. This is supported by additional findings demonstrating that the incubation of cerebellar tissue slices with crude HEM increased the covering of NF-200^+^-neuronal fibers with MBP^+^-OL cellular processes. In addition that the previous studies have characterized the significant effects of HeA and HeS in reducing amyloid-β deposition and increasing insulin-degrading enzyme in the cerebral cortex^20,21^. we here present another novel biological function of HeA and HeS in the stimulation of OL maturation. Given that HEM is enriched in HeA, the effect of HEM in stimulating OL maturation is likely due to the action of HeA. Nevertheless, HEM-induced OL maturation in vitro and in vivo could also be attributed in part to the function of HeS in the maturation of OLs.

We have noticed that exposure to HEM, HeA, or HeS increased MBP protein levels in OLs. It has been reported that a decline in MBP protein production was found in conditional ERK-knockout OLs^26,27^. ERK44/42 activation has been found to be positively correlated with active remyelination in the adult mouse corpus callosum^26^. Despite that treatment with HeA increased ERK44/42 phosphorylation in PC-12 cells to enhance NGF-induced neurite outgrowth^14^, we found that exposure of OLs to HeA for 3 days had no effect on the levels of pERK44/42 phosphorylation in OLs (Fig. S4A). Alternatively, our study showed that a 3-day exposure to HeS caused the phosphorylation of AKT to an increased trend when compared to that detected in the corresponding control groups (Fig. S4B). A number of findings has indicated that AKT acts as a positive regulator of OPC differentiation to OLs, myelination, and myelin thickness^28–30^, as well as the upregulation of MBP gene expression in lithium chloride-treated mixed glial cells^31^. Thus, it is possible that AKT signaling pathway might play the regulatory role in HEM-induced stimulation of OL maturation.

Evidence obtained by examining the overlap between MBP immunoreactivity and NF200^+^- fibers in an ex vivo cerebellar slice system indicates that crude HEM, HeA, and HeS can promote the covering of neuronal fibers with OLs. The addition of crude HEM and HeA might have induced NGF production in the cerebellar slice system, since HEM and its components (HeA-H) have been found as potent stimulators that induce NGF synthesis^15^. However, we found that NGF gene expression in OL cultures was not affected by exposure to HEM or HeA (data not shown). NGF has been reported to inhibit OL differentiation and myelination via the induction of Nogo receptor-interacting protein (LINGO-1), an axonal inhibitor of OL differentiation^32^. Thus, other undefined factor (s) produced in the cultures treated with HEM or HeA play the stimulating role in the ensheathment of neuronal fibers in cerebellar tissue culture treated with HEM, HeA, and HeS.

Considering that the chemical properties of HeA and HeS allow them to pass through the blood–brain barrier^15,33^, the increased intensity for MBP in the corpus callosum following the oral administration of HEM to neonatal rat pups could be due to the active activity of HeA and HeS in OL maturation. Cumulative evidence from animal studies has shown because of its neuroprotective properties, HeA-enriched HEM can reduce neuronal cell death and neurological symptoms in several neurological diseases, including cerebral ischemia, AD, PD, and depression^15^. The present study sheds light on the potential application of HEM and its two active compounds (HeA and HeS) in demyelinating disorders, such as multiple sclerosis. Further preclinical studies to characterize the efficacy of HEM extract and HeA/HeS in remyelination and myelin repair should be conducted in the future.

## Materials and methods

### Preparation of HEM, HeA, HeC, and HeS

Crude HEM was prepared by Grape King Biotechnology Inc. (Zhong-Li, Taiwan) by ultra-sonication of *H. erinaceus* powder in 95% ethanol (EtOH) for 2 h. The reflux solution was filtered to remove large particles and concentrated under vacuum. A H_2_O/ethyl acetate (EtOAc) solution (1:1) was used to differentiate the H_2_O layer and the EtOAc layer from the crude HEM. Silica gel column chromatography (70–230 mesh, 70 × 10 cm) was used to analyze the EtOAc layer, and n-hexane/EtOAc (3:2) was used to perform gradient separation. Sephadex LH-20 column chromatography and a subsequent silica column chromatography step were performed to obtain HeS. The n-hexane/EtOAc (1:2) fraction was then sequentially eluted with 70% MeOH through a Sephadex LH-20 column, followed by RP-18 with 60–90% MeOH to obtain HeA and HeC. The three small compounds were identified using HPLC/electrospray ionization (ESI) with mass spectrometric analysis as previously described^25^. Crude HEM was much more enriched in HeA (approximately 15 mg/g) than HeC (~ 1 mg/g) or HeS (~ 1 mg/g). HEM, HeA, HeC, and HeS were dissolved in DMSO for use with the culture system, whereas HEM was dissolved in soybean oil for the in vivo experiment. The chemical structures of HeA, HeC, and HeS are shown in Fig. 1A.

### Animals

Pregnant Sprague–Dawley (SD) rats (approximately 400 ± 20 g per animal; n = 6; RRID: RGD_728193) and male SD rat pups at postnatal day 3 (P3; n = 12) or P7 (n = 6) were purchased from BioLASCO Taiwan Co., Ltd. To prepare primary cell cultures, the animals were sacrificed by an intraperitoneal (i.p.) injection of Zoletil 50 (Virbac Taiwan Co., Ltd.; 0.1 ml/100 g for rats) before the embryos were removed. The choice of anesthetics and the method of anesthesia were carried out in compliance with the ARRIVE guidelines.

Given that MBP expressing in OLs can be detected initially at P3 in the corpus callosum at P3 following myelinated axons seen in P10^8^, rat pups (n = 6) at P3 received the daily oral administration of crude HEM (0.1 and 1.0 mg/kg) for 7 days. Since the crude HEM used for the animal experiments was dissolved in soybean oil, rat pups that received only soybean oil were used as the control group (n = 6). The pups were housed in cages with mother rats under temperature- and humidity-controlled conditions with free access to food and water. The animals were sacrificed as described above to examine myelin and OLs in the corpus callosum. The decision in the amount of animals used for the study was following the 3Rs (Replacement, Reduction and Refinement) to avoid unnecessary sacrifice. Animal use followed the National Institutes of Health (NIH) Guidelines for Animal Research (Guide for the Care and Use of Laboratory Animals) and had been approved by the National Cheng Kung University Institutional Animal Care and Use Committee, Tainan, Taiwan (IACUC approval 107,265).

### Preparation of rat OPCs

OPCs from 14.5-day-old embryos collected from pregnant rats anesthetized by an intraperitoneal injection (i.p.) of Zoletil 50 were prepared following a previously described protocol^34^. The cortical tissues were dissected from the embryonic brains and passed through a 40-μm filter. The cells were replated onto the tissue culture dishes and cultured for 5–7 days in oligosphere medium consisting of Dulbecco's modified Eagle's medium/F12 medium (DMEM/F12) (Thermo Fisher), 1% penicillin–streptomycin (P/S; Thermo Fisher), 2% B27 supplement (Thermo Fisher), 1% N2 supplement (Thermo Fisher), 10 ng/ml epidermal growth factor (Cell Guidance Systems), 10 ng/ml fibroblast growth factor-2 (Cell Guidance Systems), and 10 ng/ml platelet-derived growth factor-AA (Cell Guidance System). After the formation of oligospheres, the OPCs were dissociated and plated onto poly-D-lysine (PDL; Sigma-Aldrich)-coated dishes in growth medium (GM) for 2 days (Fig. 1B), and then maintained for 5 days in OL differentiation medium (DM) containing DMEM (Thermo Fisher), 1% P/S (Thermo Fisher), 1 mM sodium pyruvate (Sigma), 0.1% bovine serum albumin (Sigma), 50 μg/ml apotransferrin (Sigma-Aldrich), 5 μg/ml insulin (Sigma-Aldrich), 30 nM sodium selenite (Sigma), 10 nM biotin (Sigma-Aldrich), 10 nM hydrocortisone (Sigma-Aldrich a), 15 nM triiodothyronine (Sigma), 10 ng/ml ciliary neurotrophic factor (Cell Guidance Systems), and 5 μg/ml N-acetyl-cysteine (Sigma-Aldrich).

### Quantitative real-time polymerase chain reaction (QPCR)

The method was followed by the procedure as previously described^35^. In brief, cDNA was generated using M-MLV reverse transcriptase (Thermo Fisher) from RNA samples isolated from the cultures, and then incubated with Fast SYBR Green Master Mix (Applied Biosystems). The specific primers used for this study are listed in Table 1. PCR amplification was conducted for 10 min at 95 °C and 40 cycles at 95 °C for 10 s, followed by annealing at 60 °C for 10 s, and extension at 72 °C for 20 s. Glyceraldehyde-3-phosphate dehydrogenase (GAPDH) mRNA expression in each culture was used as an internal control. The cycle threshold (Ct) fluorescence values were analyzed through StepOne software v2.1 (Applied Biosystems). The expression levels of the target genes relative to the internal control are presented as 2 − ΔCT, where ΔCT = (Ct target-Ct GAPDH).

**Table 1:** The specific primer sequences for QPCR.

Gene	Sequence
Rat Olig1 (NM_021770)	Forward (5′ → 3′): GAGGGGCCTCTTTCCTTGTC Reverse (5′ → 3′): ACCGAGCTTCACAAGCCTAC
Rat Olig2 (NM_001100557)	Forward (5′ → 3′): GCTTAACAGAGACCCGAGCC Reverse (5′ → 3′): GTGGCGATCTTGGAGAGCTT
Rat Mbp (NM_001025291)	Forward (5′ → 3′): GTGGGGGTAAGAGAAACGCA Reverse (5′ → 3′): CGAACACTCCTGTGGAACGA
Rat Plp1 (NM_030990)	Forward (5′ → 3′):GGCGACTACAAGACCACCAT Reverse (5′ → 3′):AATGACACACCCGCTCCAAA
Rat GAPDH (NM_017008)	Forward (5′ → 3′): TCTACCCACGGCAAGTTC Reverse (5′ → 3′): GATGTTAGCGGGATCTCG

### Cell viability assay

OPCs were seeded at a density of 5 × 10^4^ per well in 24-well plates in GM. After treatment with HEM, HeA, HeC, and HeC, the cultures were incubated with MTT (3-(4,5-dimethylthiazol-2-yl)-2,5-diphenyltetrazolium bromide) solution (5 mg/ml; Sigma) for 4 h. The crystalized MTT products in the cultures were dissolved by the addition of dimethyl sulfoxide (Merck Millipore, Burlington, MA, USA) at 37 °C^35^, and then the absorbance at 595 nm was measured using an ELISA microplate reader (Tecan, Zurich, Swiss).

### Immunofluorescence analysis of cultured cells

The protocol used to identify OLs in the cultures was followed by the method described previously^35^. For GC immunostaining, the cells were fixed with 4% paraformaldehyde (PFA) for 10 min and treated in PBS containing anti-GC antibody (Table 2) and 1% horse serum (HS; Thermo Fisher) at 4 °C overnight. For immunostaining of MBP and neurofilament-200 (NF-200), after fixation of the cells with 4% PFA, the cultures were treated with 0.3% Triton X-100 in PBS for 30 min and incubated with primary antibodies (Table 2) in PBS containing 1% HS overnight at 4 °C. The cells were reacted with appropriate biotinylated secondary antibodies for 1 h at room temperature and avidin-Cy3 (or avidin-Alexa 488; Table 2) for another 45 min. The cultures were then subjected to 4′,6-diamidino-2-phenylindole (DAPI; Thermo Fisher) nuclear counterstaining.

**Table 2:** The primary antibodies used in this study.

Antibodies	Manufacturer	Working dilution
Polyclonal rabbit anti-phospho-Akt (Ser473)	Cell Signaling Technology RRID: AB_329825	1:1000 (WB)
Polyclonal rabbit anti-AKT	Cell Signaling Technology RRID: AB_329827	1:1000 (WB)
Monoclonal mouse anti-APC (CC1)	Merck Millipore RRID: AB_2057371	1:200 (IF)
Monoclonal mouse anti-NG2	Thermo Fisher Scientific RRID: AB_2533306	1:200 (IF)
Monoclonal mouse anti-GAPDH	Merck Millipore RRID: AB_2107445	1:2000 (WB)
Polyclonal rabbit anti-Olig2	Merck Millipore RRID: AB_570666	1:200 (IF)
Monoclonal mouse anti-GC	Merck Millipore RRID: AB_94857	1:200 (IF)
Polyclonal rabbit anti-p44/42 MAPK (ERK44/42)	Cell Signaling Technology RRID: AB_330744	1:1000 (WB)
Polyclonal rabbit anti-Phospho-p44/42 Map Kinase (Thr202/Tyr204) (p-ERK44/42)	Cell Signaling Technology RRID: AB_331646	1:1000 (WB)
Monoclonal mouse anti-MBP	Merck Millipore RRID: AB_2140491	1:200 (IF)
Polyclonal rabbit anti-PLP	Abcam RRID: AB_776593	1:2000 (WB)
Monoclonal mouse anti-MBP	Biolegend RRID: AB_2616694	1:1000 (WB)
Polyclonal-chicken anti-Neurofilament (NF200)	Merck Millipore RRID: AB_11212161	1:200 (IF)
Biotinylated anti-mouse IgG	Vector Laboratories RRID: AB_2313581	1:200 (IF)
Biotinylated anti-rabbit IgG	Vector Laboratories RRID: AB_2313606	1:200 (IF)
Biotinylated anti-Chicken IgY	Abcam RRID: AB_954958	1:200 (IF)
HRP-conjugated anti-mouse IgG	Jackson ImmunoResearch RRID: AB_10015289	1:2000 (WB)
HRP-conjugated anti-rabbit IgG	Jackson ImmunoResearch RRID: AB_2337938	1:2000 (WB)
Cy3- Streptavidin	Thermo Fisher Scientific Cat# 434,315	1:800 (IF)
Alexa Fluor 488- Streptavidin	Thermo Fisher Scientific RRID: AB_2336881	1:200 (IF)
Rhodamine-conjugated anti-mouse IgG	Thermo Fisher Scientific RRID: AB_228308	1:200 (IF)
Alexa Fluor 594-conjugated anti-rabbit IgG	Abcam RRID: AB_2782993	1:200 (IF)

*IF* immunofluorescence, *IHC* immunohistochemistry, *WB* western blot.

### Brain tissue section preparation and immunofluorescence

After the animals had been sacrificed as described above, the brain tissues were removed, postfixed with 4% PFA overnight, and cryoprotected in 30% (w/v) sucrose in PBS^35^. The brains were embedded in Tissue-Tek optimal cutting temperature compound (Electron Microscopy Sciences, Torrance, CA, USA) and sliced into the coronal sections with a 20-μm thickness. The brain tissue slices were collected in PBS for use. The freely floating tissue sections containing corpus callosum lining around lateral ventricle and caudate nucleus were selected and treated with 1% Triton X-100 in PBS at 4 °C overnight and incubated with primary antibody at a dilution of 1:200 in PBS containing 0.1% Triton X-100 and 1% HS at 4 °C overnight. The tissues were subsequently incubated with the appropriate biotinylated secondary antibodies (Table 2) for 1 h followed by Alexa 488 or Cy3-avidin for 45 min. DAPI nuclear counterstaining was then performed. The immunostained cells were visualized under a Nikon E800 epifluorescence microscope equipped with a CCD camera and an Olympus FluoView laser-scanning confocal microscope (FV1000, Japan).

### Western blot analysis

The total protein was extracted from the cultures using a lysis buffer containing 1% NP-40, 1% Triton X-100, 1% SDS, and a protease inhibitor cocktail (Thermo Fischer). As previously described^35^, the protein samples (100 μg/sample) were loaded onto a 12.5% SDS polyacrylamide gel. After electrophoresis, the proteins were transferred to a nitrocellulose membrane and immunoblotted overnight at 4 °C with primary antibodies (Table 2). The immunoblotted membrane was incubated with secondary antibodies conjugated with peroxidase for 60 min at RT. The signal was detected by chemiluminescence using the ECL-Plus detection system (PerkinElmer Life Sciences).

### Evaluation of oligodendrocyte differentiation in vitro

To evaluate OL differentiation, the morphology of cells expressing OL lineage markers (i.e., GC and MBP) was examined using an epifluorescence microscope with a 20 × objective lens to capture a microscopic image with the size of 0.23 mm^2^, which contained approximately 150–200 cells per images^35^. OLs expressing GC and MBP in each image were quantified using NIH ImageJ analysis software version 1.52a (RRID: SCR_003070; https://imagej.nih.gov/ij/)^36^. The experiments were repeated with the three independent cell preparations. The results are presented as a percentage of the values obtained from cell cultures treated with Vehicle and HEM (or HeA, HeC, or HeS). Western blot analysis of the measurement of myelin proteins (i.e., MBP and PLP) was also conducted to validate the results from cell quantification.

### Cerebellar slice culture

The survival rate of neurons in the cerebellar slices isolated from P7–P10 rodents was better than those prepared from the age of P1–P5^37^. Thus, the cerebellum was taken from P7 rats and sectioned into fine sagittal vermal slices with a 200-μm thickness using a Microslicer DTK-1000 vibratome as previously described^38^. The four cerebellar tissue slices were then laid onto a Millicell-CM culture insert (Millipore, 0.4 μm) and maintained in slice culture medium (50% MEM with Earle's salts, 35% Earle's balanced salt solution, and 15% heat-inactivated HS) at 37 °C for 3 days. HEM, HeA, HeC and HeS at different concentrations were then added to the cultures, which were incubated for 7 days. The culture media were replaced every 2–3 days with fresh medium containing HEM, HeA, HeC, or HeS. The cerebellar slices were fixed with 4% PFA and permeabilized by 1% Triton X-100 in PBS, followed by immunofluorescence for MBP and NF200.

### Statistical analysis

Data analyses were performed using the one-way analysis of variance (ANOVA) test with Benjamini and Hochberg false discovery rate (FDR) correction, and presented as the means ± SEMs of the three independent cell preparation or at least three independent ex vivo experiments. In all comparisons, differences were considered statistically significant at *p* < 0.05.

### Ethics approval

Animal use followed the Guidelines for Animal Research (Guide for the Care and Use of Laboratory Animals) approved by the National Cheng Kung University Institutional Animal Care and Use Committee, Tainan, Taiwan.

## Supplementary information

Supplementary information.

## Abbreviations

APC: Adenomatous polyposis coli
CNS: Central nervous system
Ct: Cycle threshold
DM: Differentiation medium
DMEM/F-12: Dulbecco's modified Eagle's medium/F12 medium
GM: Growth medium
*H.*: *Hericium*
HeA: Erinacine A
HeC: Erinacine C
HeS: Erinacine S
HEM: *H. erinaceus* Mycelium
MBP: Myelin basic protein
MTT: 3-(4,5-Dimethylthiazol-2-yl)-2,5-diphenyltetrazolium bromide
NF200: Neurofilament-200
OL: Oligodendrocyte
OPC: Oligodendrocyte precursor cell
PFA: Paraformaldehyde
SD: Sprague–Dawley
PDL: Poly-D-lysine
RRID: Research Resource Identifier (see scicrunch.org)
