# Author Correction: *Hericium erinaceus* mycelium and its small bioactive compounds promote oligodendrocyte maturation with an increase in myelin basic protein

**DOI:** 10.1038/s41598-021-88168-w

**Published:** 2021-04-21

**Authors:** Hui-Ting Huang, Chia-Hsin Ho, Hsin-Yu Sung, Li-Ya Lee, Wan-Ping Chen, Yu-Wen Chen, Chin-Chu Chen, Chung-Shi Yang, Shun-Fen Tzeng

**Affiliations:** 1 Department of Life Sciences, College of Bioscience and Biotechnology, National Cheng Kung University, #1 University Road, Tainan, Taiwan; 2 Grape King Biotech Research Institute, Zhongli, 320 Taiwan; 3 Institute of Biomedical Engineering and Nanomedicine, National Health Research Institutes, Zhunan Town, Miaoli County, Taiwan

Correction to: *Scientific Reports* 10.1038/s41598-021-85972-2, published online 22 March 2021

The original version of this Article contained an error in Figure 3 where the labels in panel D were incorrect. As a result,

“mg/ml”

now reads:

“mg/kg”

Additionally,

“μg/ml”

now reads:

“mg/kg”

Furthermore, in the legend of Figure 3,

“Scale bar in (**A**) 20 μm; in (**B**) 50 μm”

now reads:

“Scale bar in (**B**) 20 μm; in (**D**) 50 μm”

The original Figure 3 and accompanying legend appear below. The original Article has been corrected.

**Figure 3:**
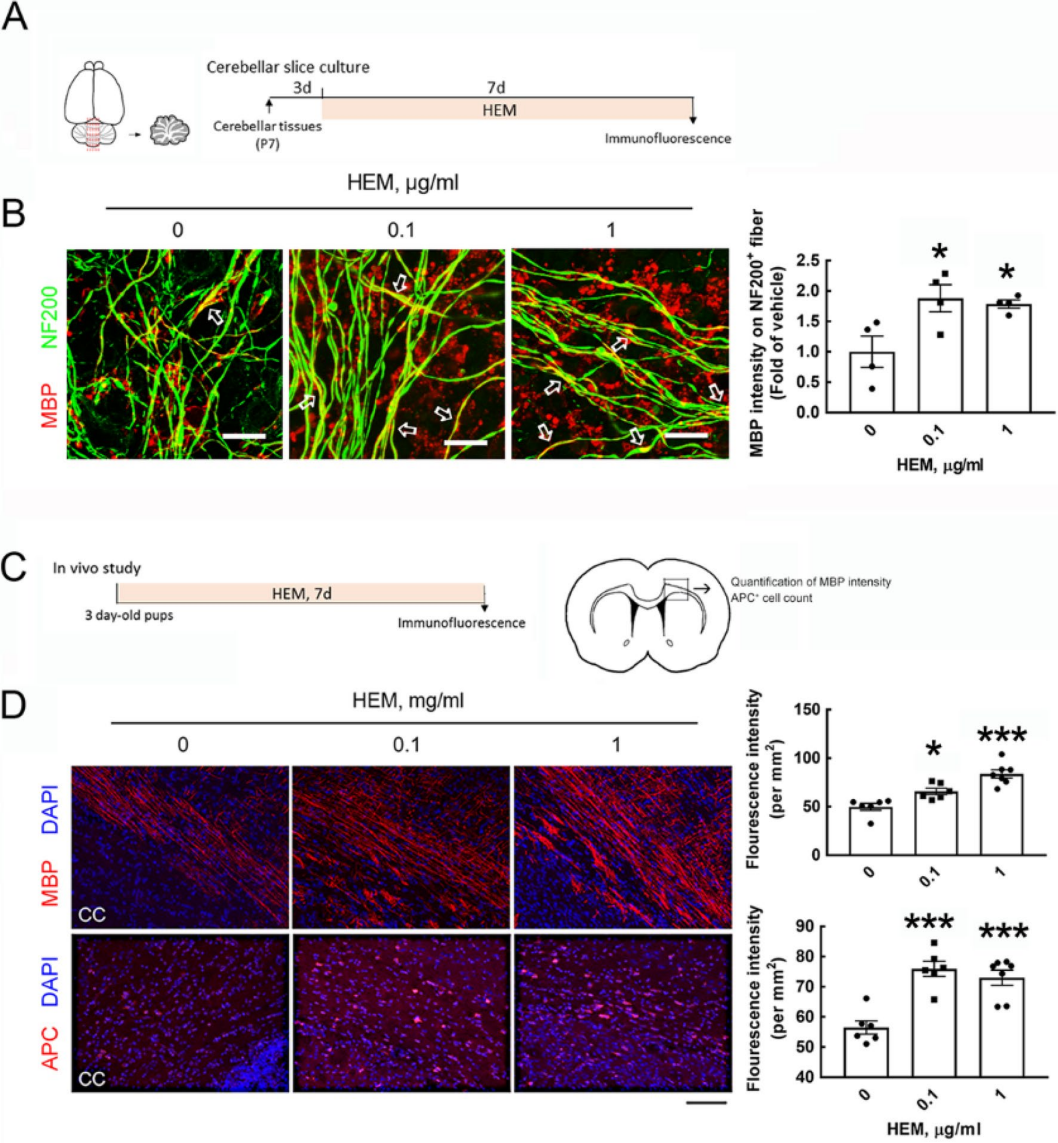
Ex vivo and in vivo experiments reveal the effect of crude HEM in stimulating MBP production and OL maturation. (**A**) Cerebellar tissue slices taken from the vermis (as indicated by red lines) were prepared from P7 rats and then cultured for 3 days. Crude HEM at concentrations of 0.1 and 1 μg/ml was added to the cultures for 7 days. The cultures were subjected to immunofluorescence for NF200 and MBP staining. (**B**) Confocal images show overlap between the immunoreactive intensity of MBP with NF200^+^-fibers (arrows), which was quantified using ImageJ software. The data were obtained from four random fields per culture (right panel). The results are presented as the means ± SEMs of the four tissue slices for each treatment. Each dot denotes data obtained from one tissue slice. (**C**) For the in vivo study, crude HEM was administered to rat pups at the age of P3 orally daily for 7 consecutive days. After brains were removed and cryostat sectioned, followed by immunofluorescence for MBP or APC. MBP intensity and APC^+^-cells were quantified in the body portion of the corpus callosum using ImageJ software. (**D**). After immunofluorescence by anti-MBP (red) or anti-APC (red), the brain tissue sections were subjected to DAPI nuclear counterstaining (blue). The representative images in the body portion of the corpus callosum were captured by confocal microscopy, and the immunoreactive intensity of MBP and APC was then quantified as described in “Materials and methods” section. The results are presented as the means ± SEMs of 6 animals in each group. *p < 0.05, ***p < 0.001 versus the vehicle-treated control culture. Scale bar in (**A**) 20 μm; in (**B**) 50 μm.

