# Supplementary material for: Hericium erinaceus mycelium and its small bioactive compounds promote oligodendrocyte maturation with an increase in myelin basic protein
Source: Sci Rep. 2021 Mar 22;11:6551. doi: 10.1038/s41598-021-85972-2 (PMC7985201; doi:10.1038/s41598-021-85972-2)
Supplement: Supplementary file 1 — Supplementary information. [file 41598_2021_85972_MOESM1_ESM.pdf]

Supplementary Materials

Hericium erinaceus mycelium and its small bioactive compounds promote oligodendrocyte maturation with an increase in myelin basic protein

Hui-Ting Huang[1], Chia-Hsin Ho[1], Hsin-Yu Sung[1], Li-Ya Lee[2], Wan-Ping Chen[2], Yu-Wen Chen[2], Chin-Chu Chen[2], Chung-Shi Yang[3], Shun-Fen Tzeng[1]*

[1]Department of Life Sciences, College of Bioscience and Biotechnology, National Cheng Kung University, Tainan, Taiwan
[2]Grape King Biotechnology Inc., Zhong-Li 320, Taiwan
[3]Institute of Biomedical Engineering and Nanomedicine, National Health Research Institutes, Zhunan, Miaoli County, Taiwan

*** To whom correspondence should be addressed**
Dr. Shun-Fen Tzeng, #1 University Road, Department of Life Sciences, National Cheng Kung University, Tainan, Taiwan
E-mail: stzeng@mail.ncku.edu.tw
Phone: 886-6-2757575 ext. 58129

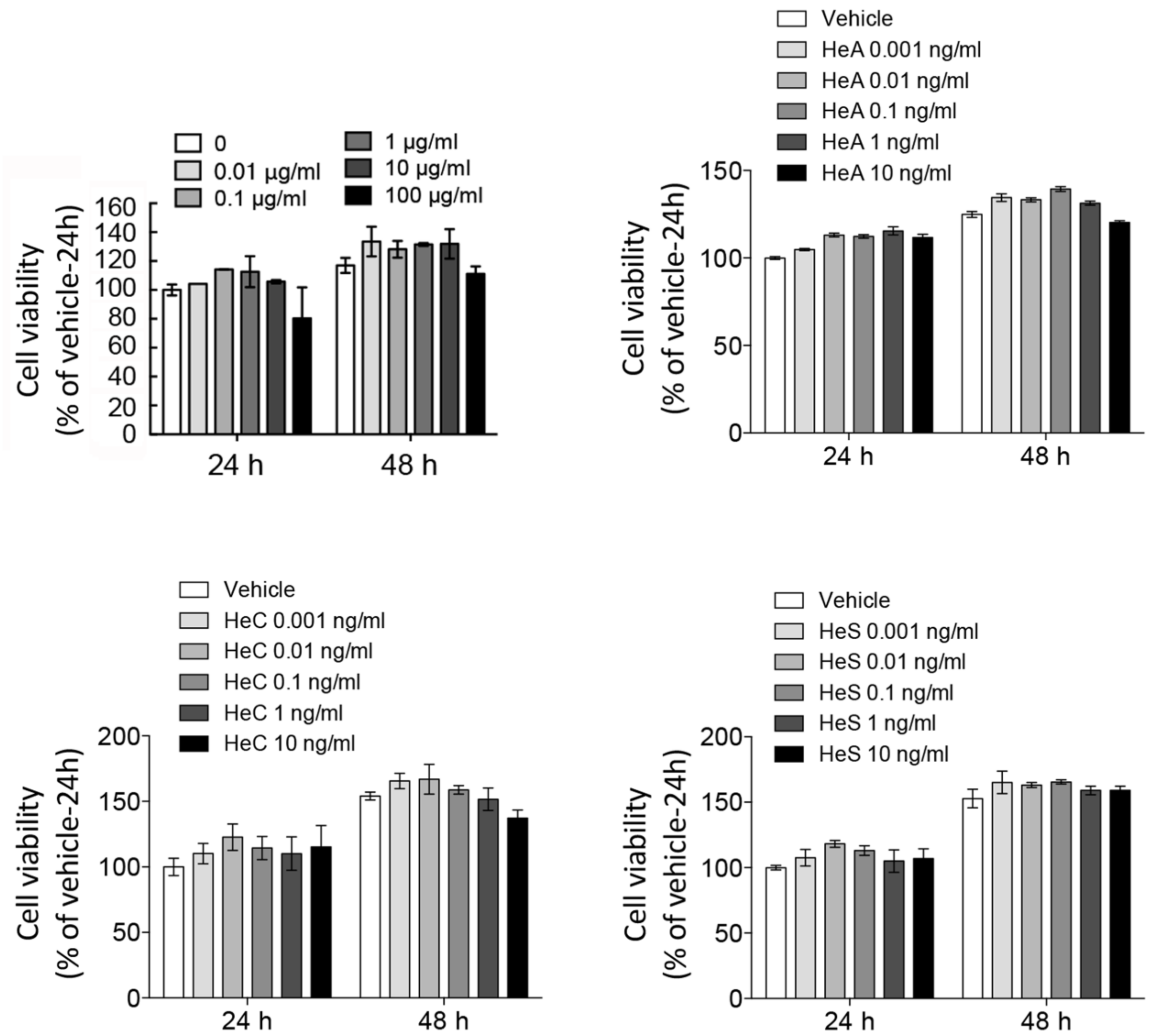

Figure S1. Effect of HEM crude, HeA, HeC, or HeS on the cell viability of OPCs. OPCs were treated with HEM crude, HeA, HeC, or HeS at the indicated concentrations in GM for 24 or 48 h. The cultures were subjected to MTT cell viability assay. The results show that exposure to HEM crude, HeA, HeC, or HeS at the indicated concentrations was not toxic to OPCs. The experiments were repeated twice with similar results.

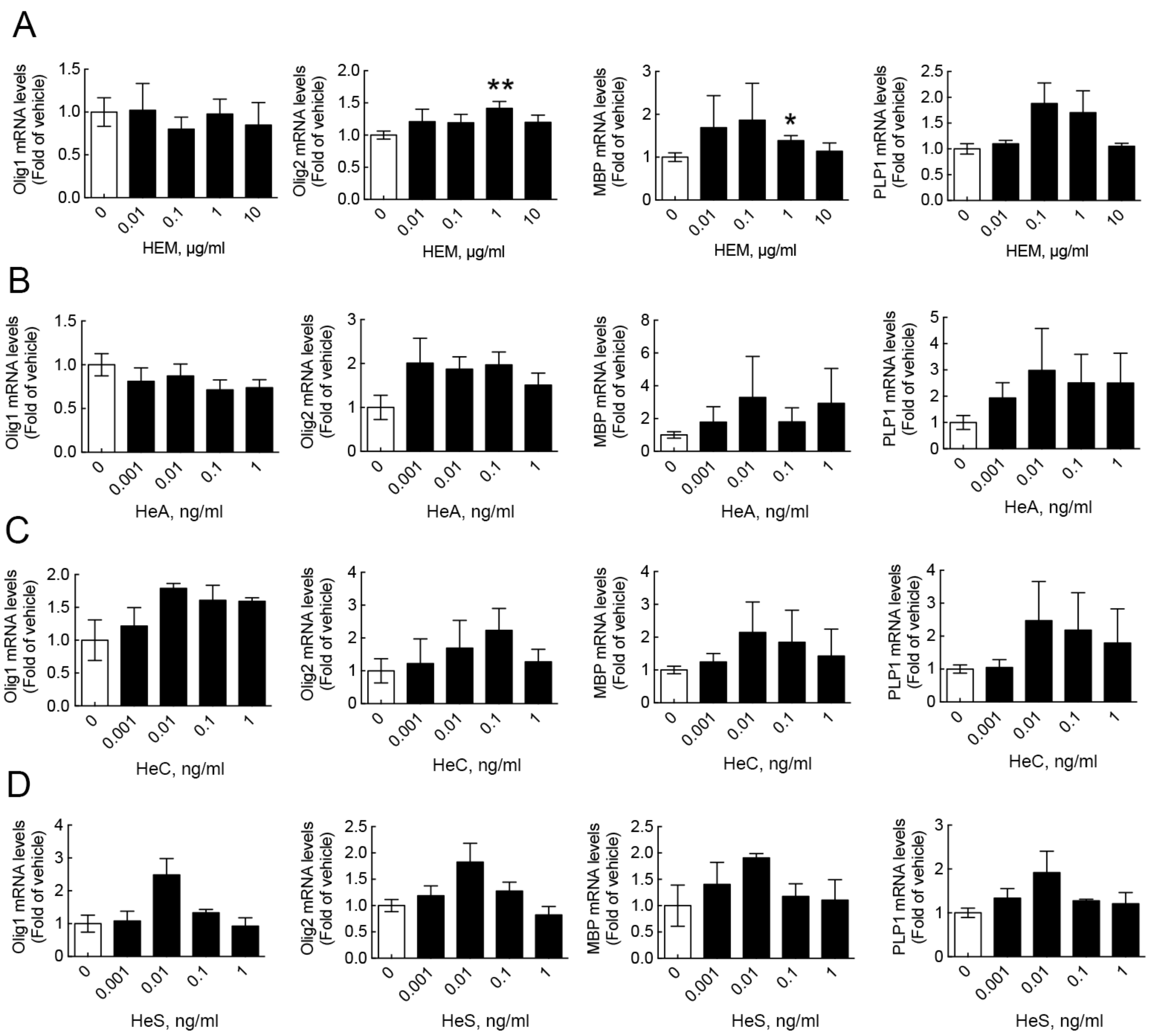
A
B
C
D
Olig1 mRNA levels (Fold of vehicle)
Olig2 mRNA levels (Fold of vehicle)
MBP mRNA levels (Fold of vehicle)
PLP1 mRNA levels (Fold of vehicle)
HEM, μg/ml
HeA, ng/ml
HeC, ng/ml
HeS, ng/ml

Figure S2. Expression of OL differentiation-associated genes after exposure of OPCs to HeA, HeC, or HeS at the different concentrations. As shown in Fig. 1B, OPCs were maintained in DM and then treated with HEM (A), HeA (B), HeC (C), or HeS (D) at the different concentrations for 3 days. RNA samples were prepared and subjected to QPCR for the measurement of OL differentiation- associated genes (i.e. Olig1, Olig2, MBP, and PLP1). The results show that HeA, HeC, and HeS did not induce significant change in these gene expression. Data are presented as the means ± SEMs of three repeated experiments.

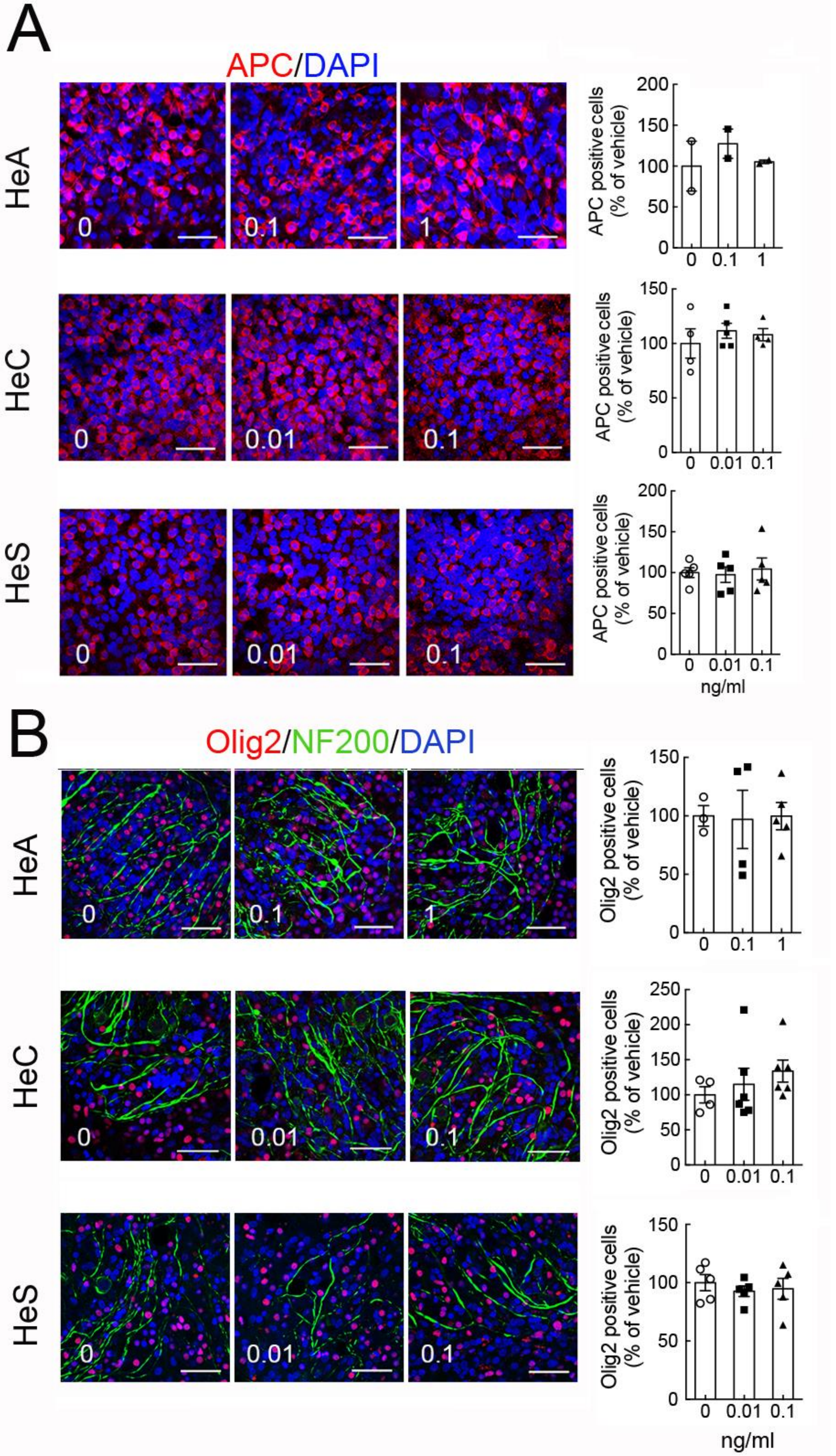

A
APC/DAPI
HeA
HeC
HeS
0
0.1
1
0.01
APC positive cells (% of vehicle)
ng/ml
B
Olig2/NF200/DAPI
Olig2 positive cells (% of vehicle)

Figure S3. No effect of HeA, HeC, or HeS on the number of APC-positive or Olig2-positive OLs in cerebellar tissue slices. HeA, HeC, and HeS at the indicated concentrations were added to cerebellar slice cultures and incubated for 7 days (Fig. 1C). A. The cultures were subjected to APC immunofluorescence (red) and DAPI nuclear counterstaining (blue). B. The cultures were subjected to immunostaining for Olig2 (red) and NF200 (green) followed by DAPI nuclear counterstaining. The representative images captured from confocal microscopy (left panel) and immunostained-positive OLs in 4 random fields per culture were quantified. Data are shown as a percentage of the values obtained from treatment with HeA, HeC, or HeS, and normalized by the data obtained from the vehicle-treated control (right panel). The results are presented as the means $\pm$ SEMs of the total tissue slices prepared from 10 animals. Each dot denotes data obtained from one tissue slice. Scale bar, 50 μm.

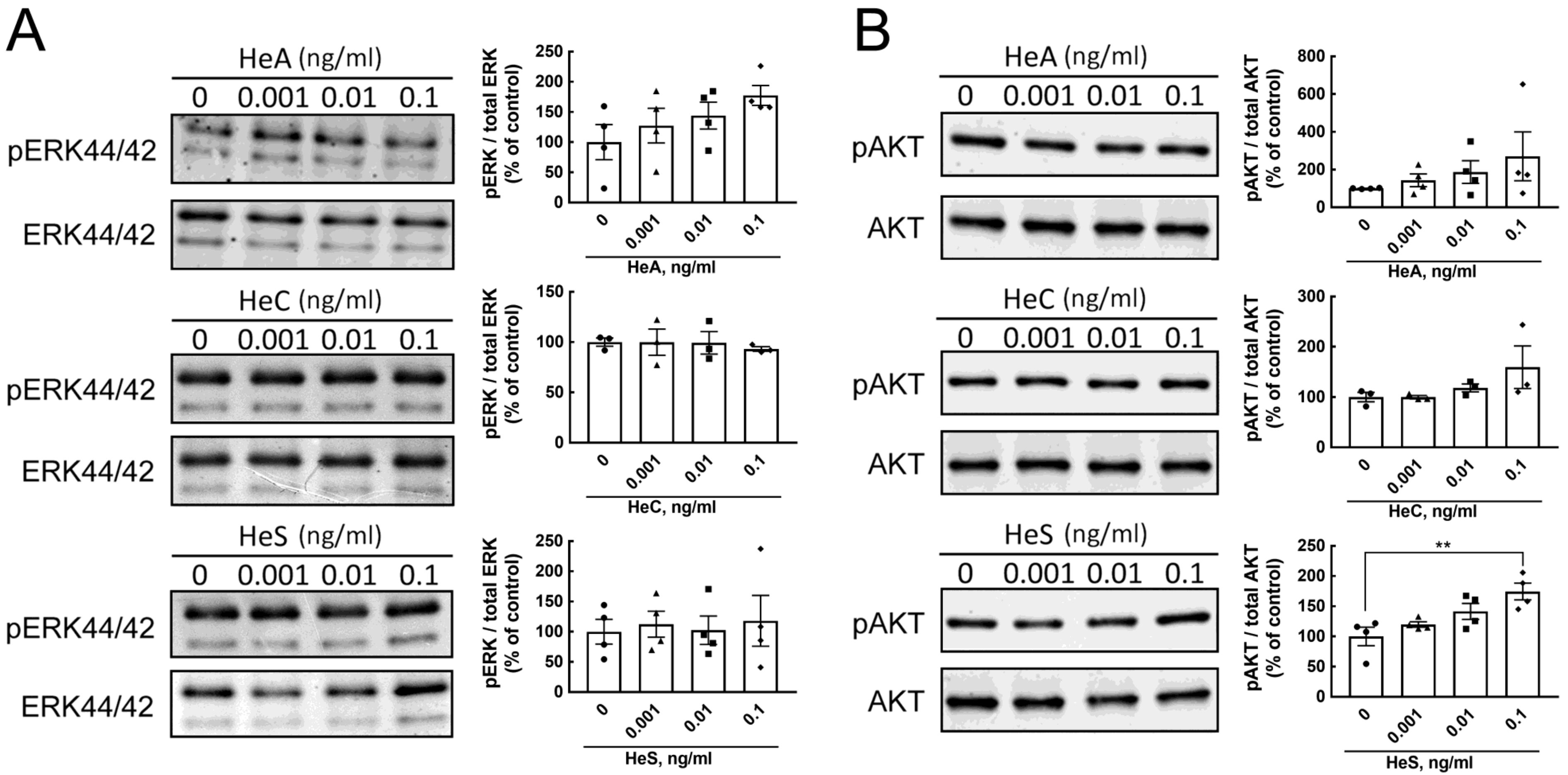

Figure S4. Effect of HeA and HeS on ERK44/42 and AKT phosphorylation in OLs. As illustrated in Fig. 1B, total proteins were isolated from OL cultures after treatment with HeA, HeC, or HeS at the indicated concentrations for 3 days and then analyzed by Western blot analysis to measure ERK44/42 (A) and AKT (B) phosphorylation. The intensity of phosphorylated ERK44/42 or phosphorylated AKT in the immunoblots was quantified by ImageJ software version 1.52a (https://imagej.nih.gov/ij/), and normalized to the level of ERK44/42 or AKT, which were used as relevant

loading controls. The results indicate that ERK44/42 phosphorylation was insignificantly increased in the presence of HeA, HeC, or HeS for 3 days. Yet, HeS caused AKT phosphorylation with an increased trend. The raw immunoblot images are shown in Fig. S9 for HeA, Fig.S10 for HeC, and Fig. S11 for HeS. Data are presented as the means ± SEMs of three or four repeated experiments. *$p<0.05$, **$p < 0.01$ compared with the control culture.

For Fig. 2B

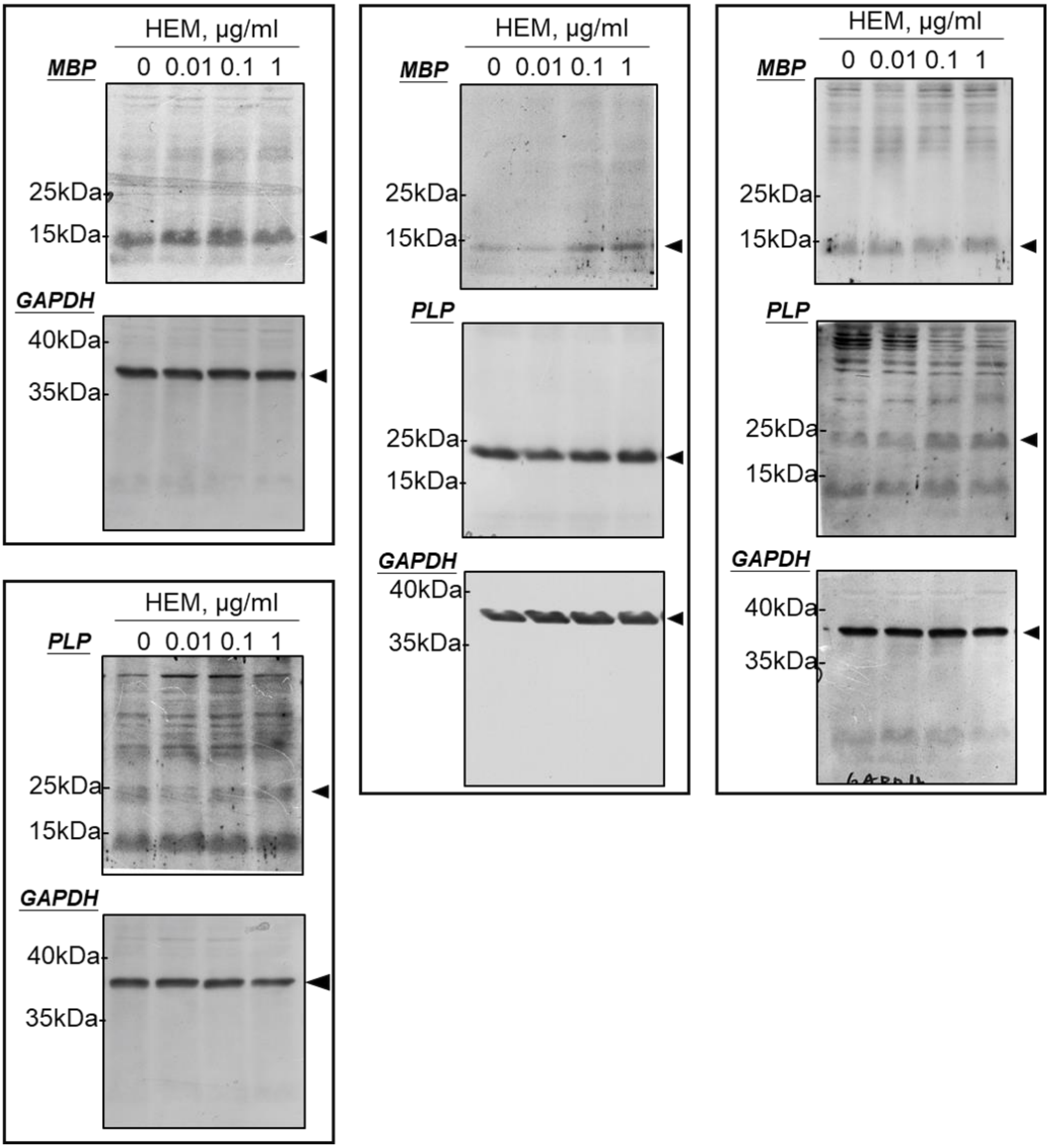

Figure S5. All of the immunoblot images used for the quantification shown in Fig. 2B. Arrowheads in the immunoblots indicate the band corresponding to relative proteins.

For Fig. 5A

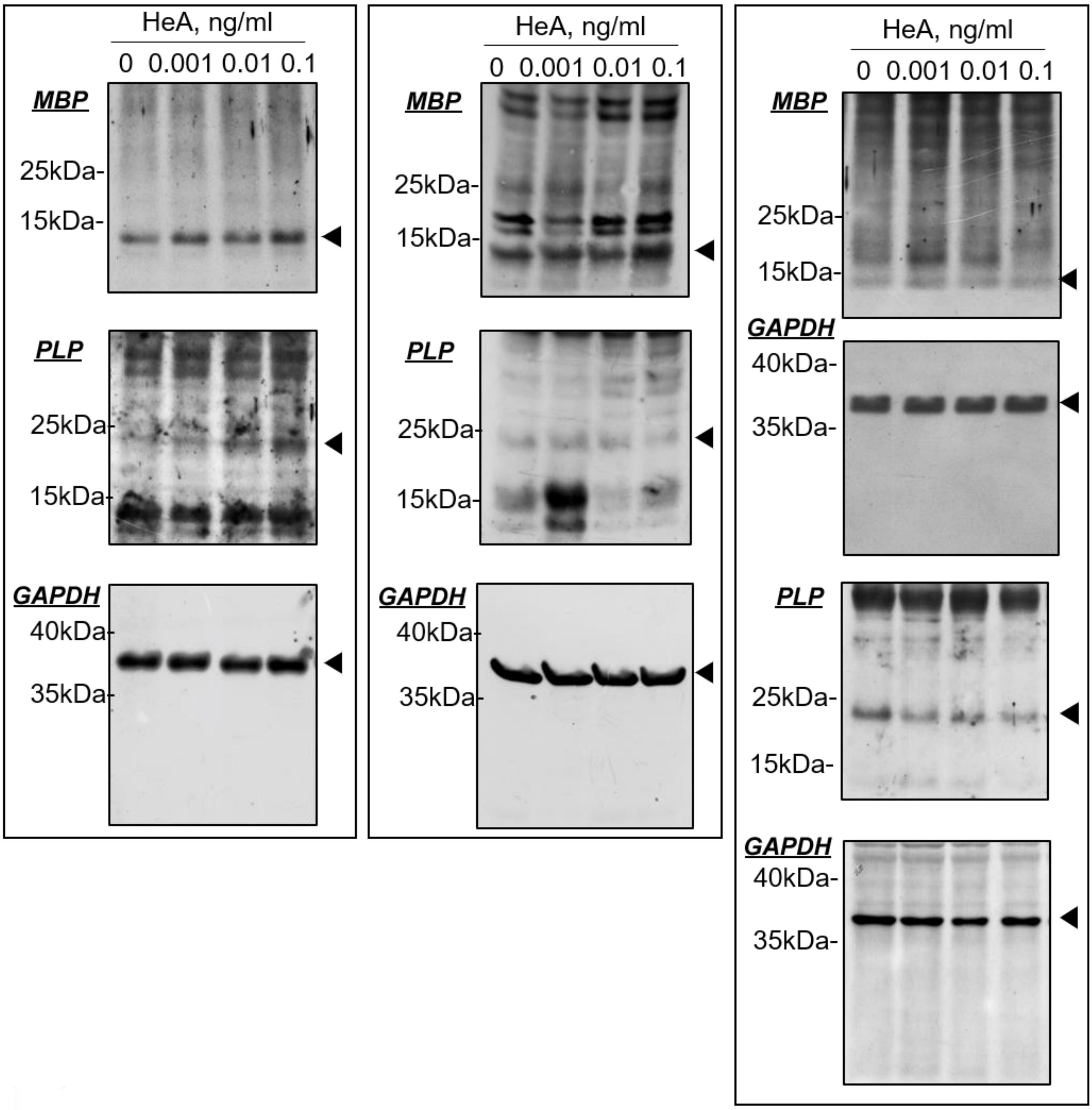

Figure S6. All of the immunoblot images used for the quantification shown in Fig. 5A. Arrowheads in the immunoblots indicate the band corresponding to relative proteins.

For Fig. 5B

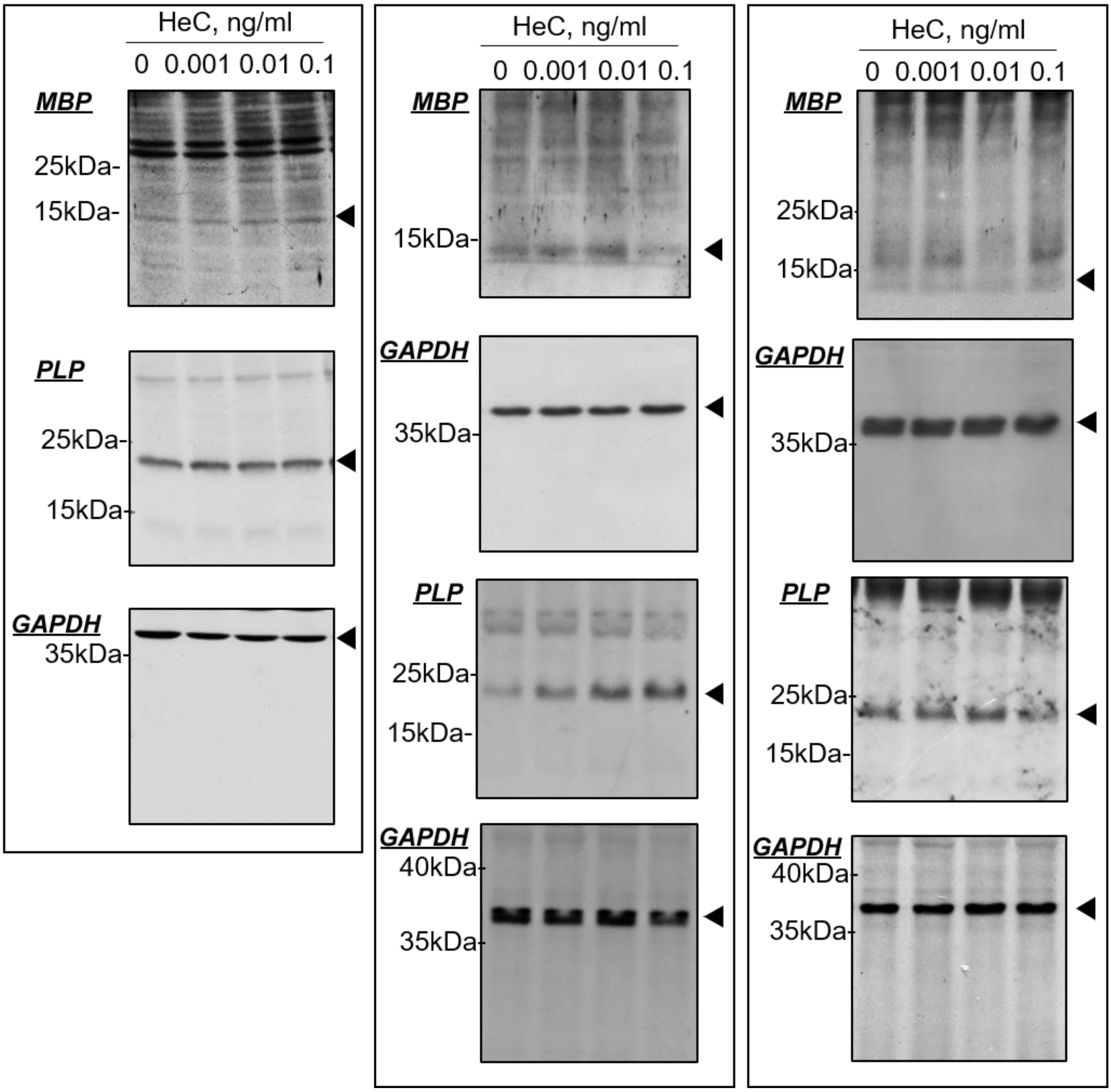

Figure S7. All of the immunoblot images used for the quantification shown in Fig. 5B. Arrowheads in the immunoblots indicate the band corresponding to relative proteins.

For Fig. 5C

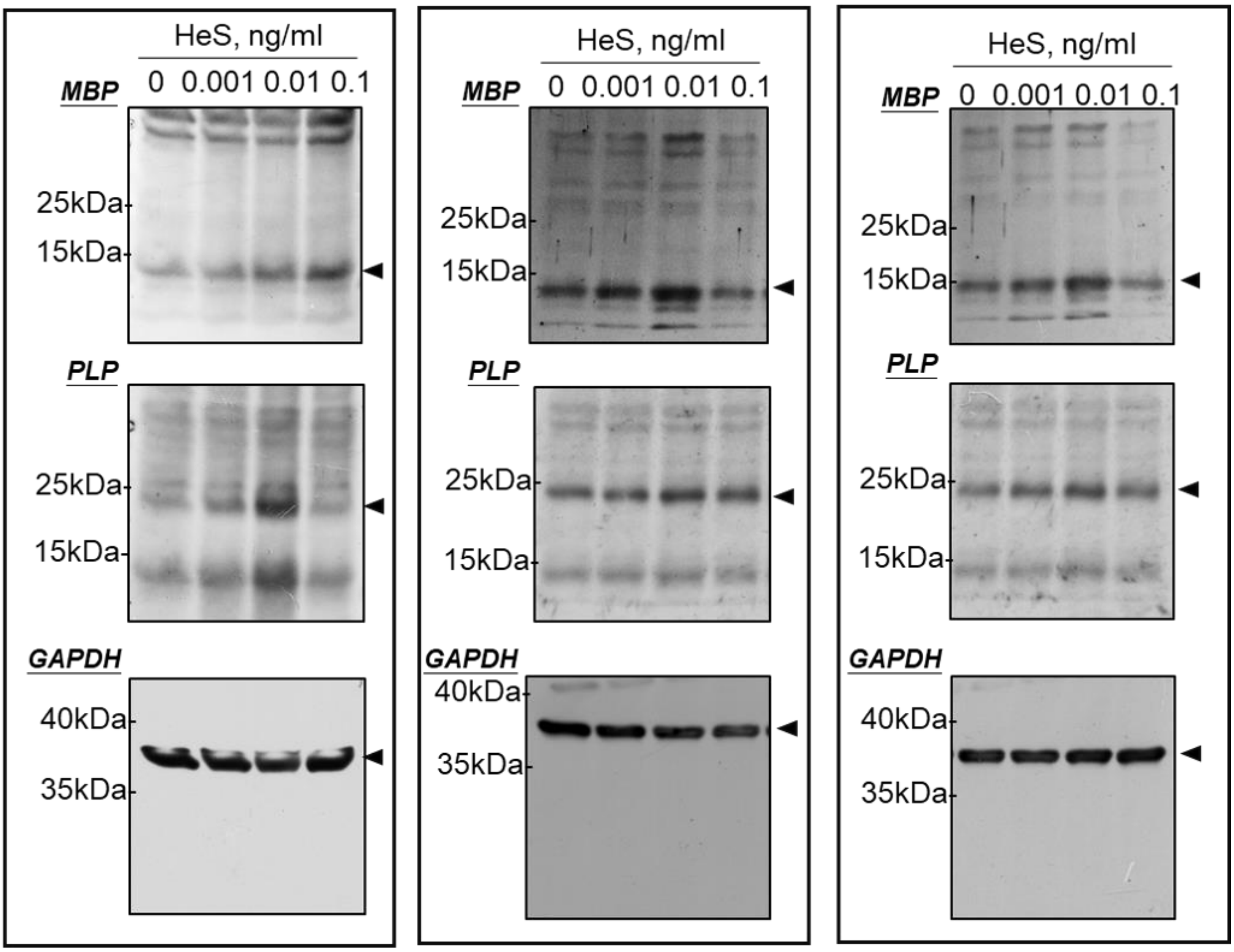

Figure S8. All of the immunoblot images used for the quantification shown in Fig. 5C. Arrowheads in the immunoblots indicate the band corresponding to relative proteins.

For HeA in Fig. S4

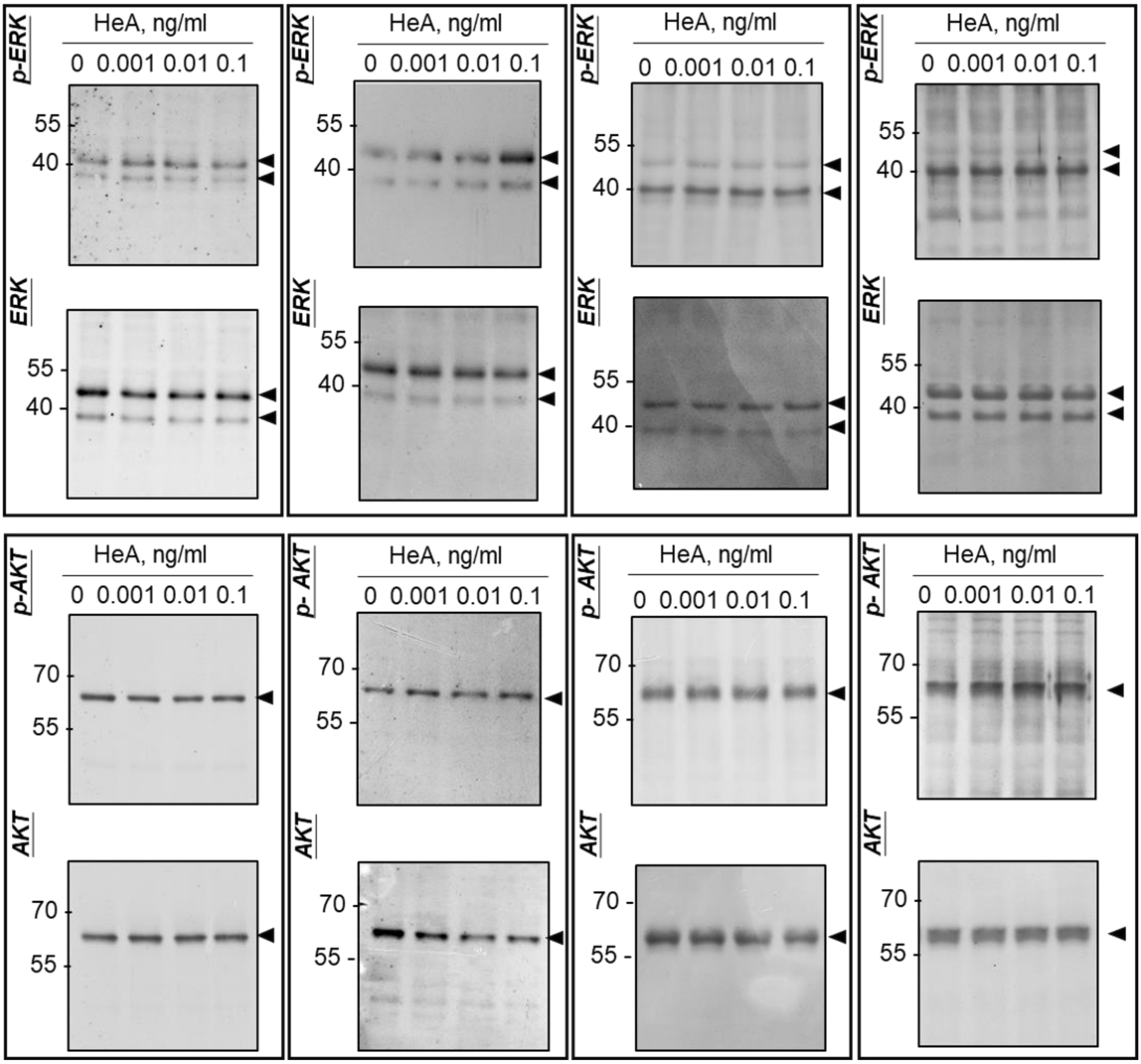

Figure S9. All of the immunoblot images used for the quantification of HeA-treated groups shown in Fig. S4. Arrowheads in the immunoblots indicate the band corresponding to relative proteins.

For HeC in Fig. S4

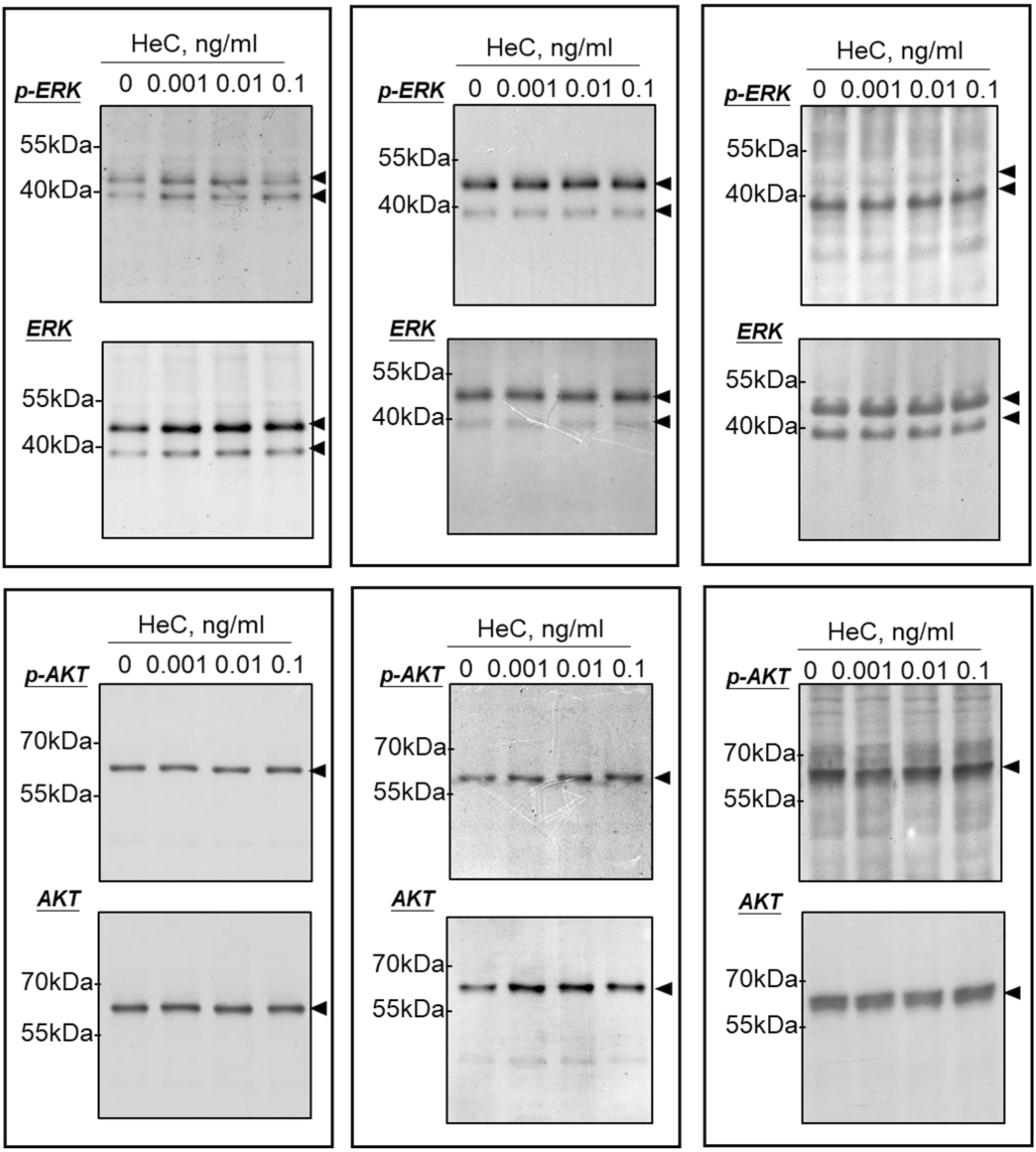

Figure S10. All of the immunoblot images used for the quantification of HeC-treated groups shown in Fig. S4. Arrowheads in the immunoblots indicate the band corresponding to relative proteins.

For HeC in Fig. S4

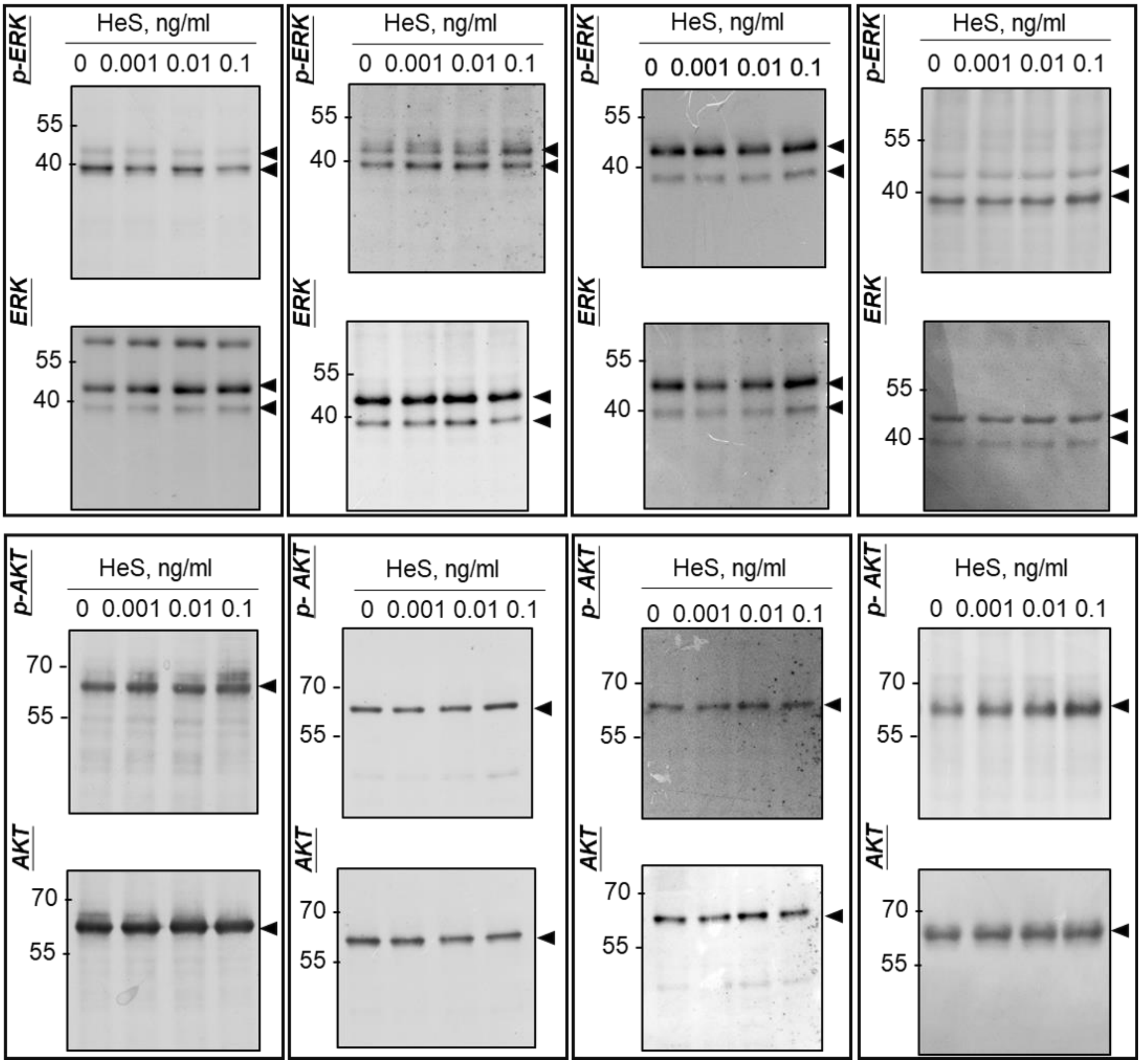

Figure S11. All of the immunoblot images used for the quantification of HeC-treated groups shown in Fig. S4. Arrowheads in the immunoblots indicate the band corresponding to relative proteins.
